# Supplementary material for: Comparisons of Videolaryngoscopes for Intubation Undergoing General Anesthesia: Systematic Review and Network Meta-Analysis of Randomized Controlled Trials
Source: J Pers Med. 2022 Feb 26;12(3):363. doi: 10.3390/jpm12030363 (PMC8954588; doi:10.3390/jpm12030363)
Supplement: Supplementary file 1 [file jpm-12-00363-s001.zip › Document S1.pdf]

Supplementary Digital File S1: Comprehensive list presenting the search strategy

(Searching time – Jan. 30, 2020)

Ovid-Medline

1. exp laryngoscopes/
2. exp laryngoscopy/
3. (video?laryngoscop* or ((video or indirect) adj3 laryngoscop*) or Airtaq or Bullard or Pentax or Glidescope or McGrath or Storz or Venner or King Vision or Vividtrac or CoPilot VL or UE scope).mp.
4. or/1-3
5. ((randomized controlled trial or controlled clinical trial).pt. or randomized.ab. or placebo.ab. or drug therapy.fs. or randomly.ab. or trial.ab. or group.ab.) not (animals not (humans and animals)).sh.
6. 4 and 5

Ovid-Embase

1. exp laryngoscope/

2. exp laryngoscopy/

3. (video?laryngoscop* or ((video or indirect) adj3 laryngoscop*) or Airtaq or Bullard or Pentax or Glidescope or McGrath or Storz or Venner or King Vision or Vividtrac or CoPilot VL or UE scope).mp.

4. or/1-3

5. ((randomized controlled trial or controlled clinical trial).pt. or randomized.ab. or placebo.ab. or drug therapy.fs. or randomly.ab. or trial.ab. or group.ab.) not (animals not (humans and animals)).sh.

6. 4 and 5

Cochrane Central Register of Controlled Trials

1. MeSH descriptor: [Laryngoscope] explode all trees

2. MeSH descriptor: [Laryngoscopy] explode all trees

3. “Airtaq”:ti,ab,kw.

4. “Bullard”:ti,ab,kw.

5. “Pentax”:ti,ab,kw.

6. “Glidescope”:ti,ab,kw.

7. “McGrath”:ti,ab,kw.

8. “Storz”:ti,ab,kw.

9. “Venner”:ti,ab,kw.

10. “King Vision”:ti,ab,kw.

11. “Vividtrac”:ti,ab,kw.

12. “CoPilot VL”:ti,ab,kw.

13. “UE scope”:ti,ab,kw.

14. #1 or #2 or #3 or #4 or #5 or #6 or #7 or #8 or #9 or #10 or #11 or #12 or #13
