# Supplementary material for: Comparisons of Videolaryngoscopes for Intubation Undergoing General Anesthesia: Systematic Review and Network Meta-Analysis of Randomized Controlled Trials
Source: J Pers Med. 2022 Feb 26;12(3):363. doi: 10.3390/jpm12030363 (PMC8954588; doi:10.3390/jpm12030363)
Supplement: Supplementary file 1 [file jpm-12-00363-s001.zip › Figure S1 w pdf.pdf]

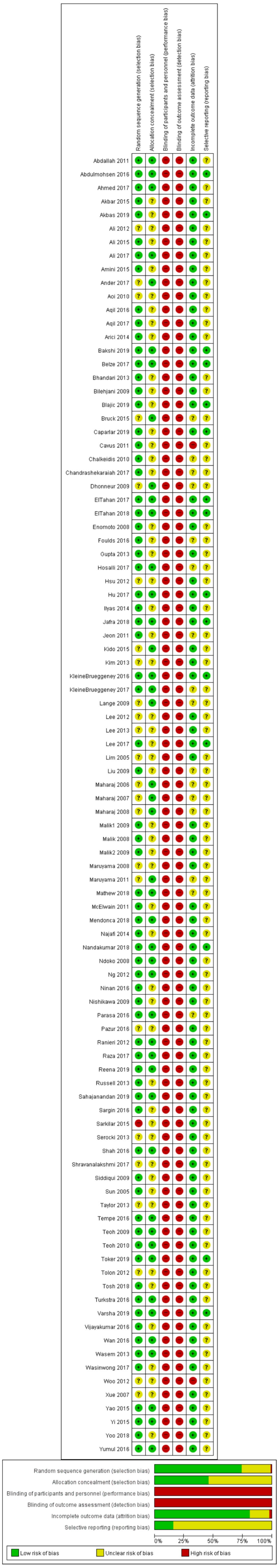

Figure S1. Risk of bias assessment. Author's judgments about each risk of bias item for each included study.
