# Supplementary material for: Comparisons of Videolaryngoscopes for Intubation Undergoing General Anesthesia: Systematic Review and Network Meta-Analysis of Randomized Controlled Trials
Source: J Pers Med. 2022 Feb 26;12(3):363. doi: 10.3390/jpm12030363 (PMC8954588; doi:10.3390/jpm12030363)
Supplement: Supplementary file 1 [file jpm-12-00363-s001.zip › Figures S2-S4.pdf]

(A)

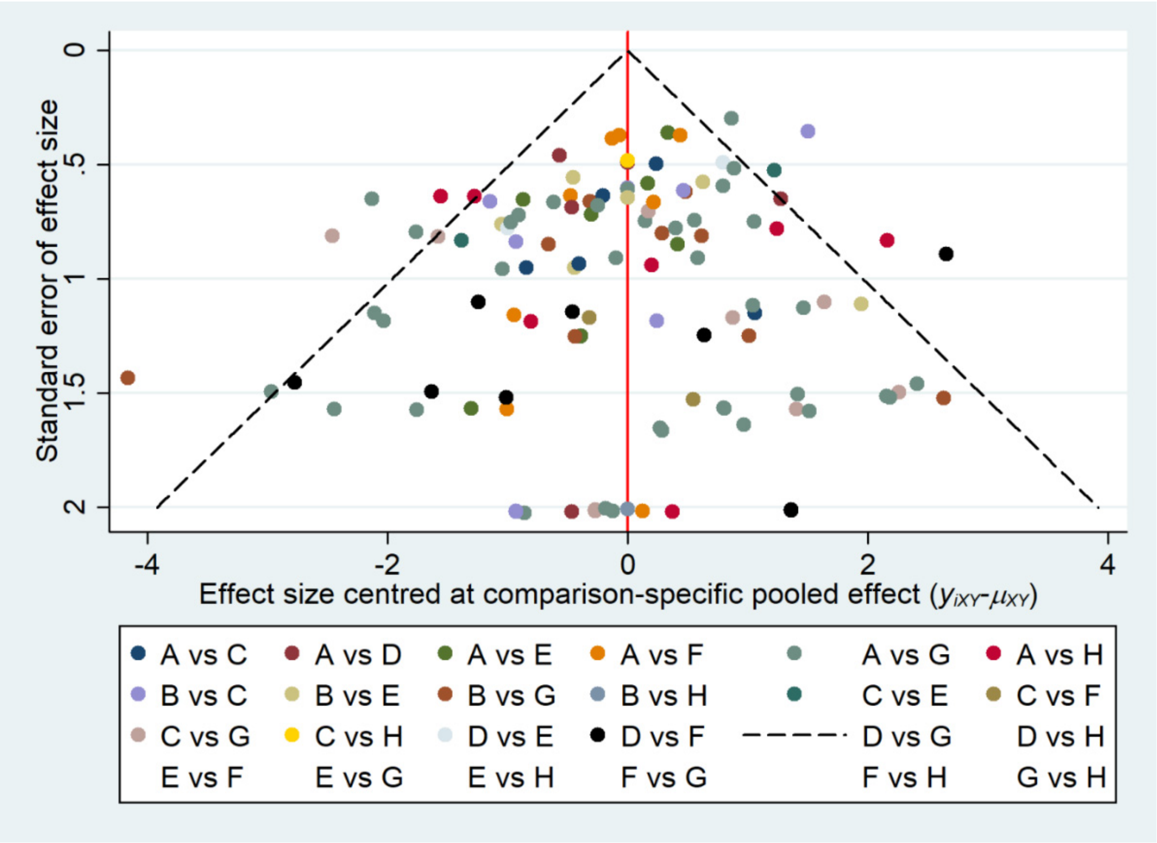
Standard error of effect size
0
.5
1
1.5
2
-4
-2
0
2
4
Effect size centred at comparison-specific pooled effect (y_{iXY}-μ_{XY})
A vs C
A vs D
A vs E
A vs F
A vs G
A vs H
B vs C
B vs E
B vs G
B vs H
C vs E
C vs F
C vs G
C vs H
D vs E
D vs F
D vs G
D vs H
E vs F
E vs G
E vs H
F vs G
F vs H
G vs H

(B)

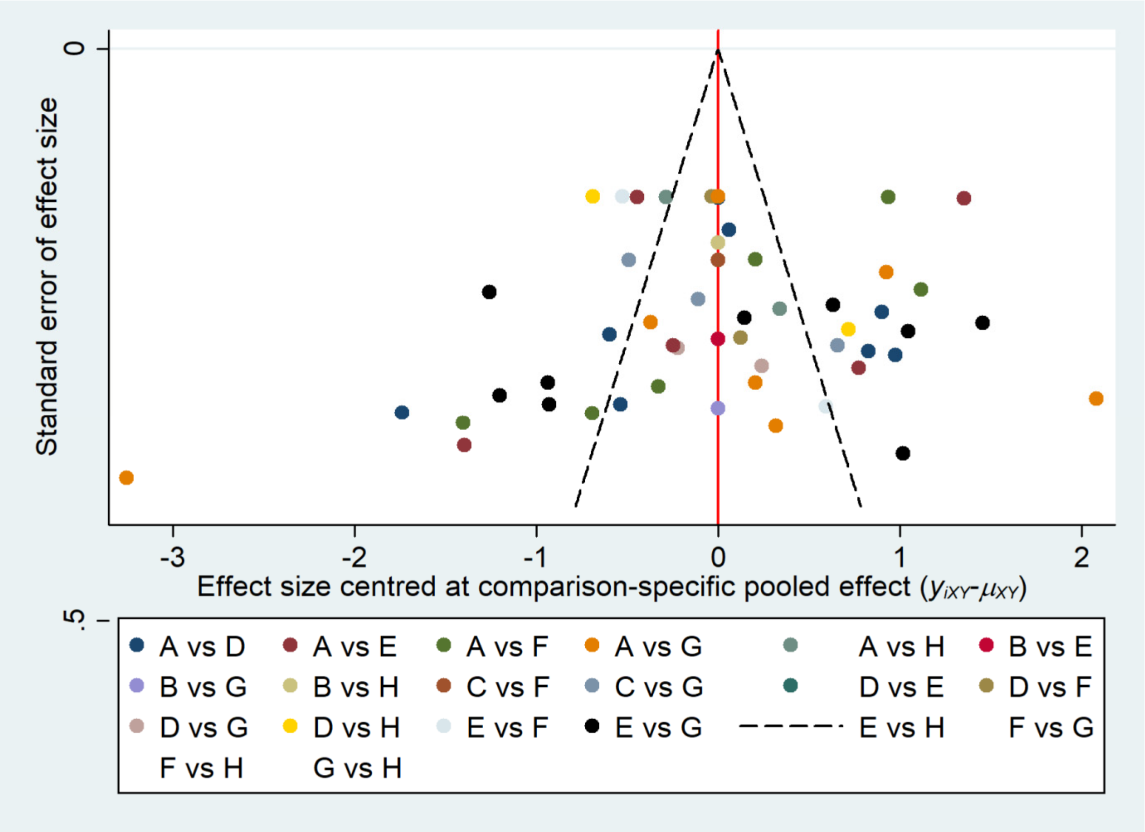
Standard error of effect size
0
.5
-3
-2
-1
0
1
2
Effect size centred at comparison-specific pooled effect (y_{iXY}-μ_{XY})
A vs D
A vs E
A vs F
A vs G
A vs H
B vs E
B vs G
B vs H
C vs F
C vs G
D vs E
D vs F
D vs G
D vs H
E vs F
E vs G
E vs H
F vs G
F vs H
G vs H

(C)

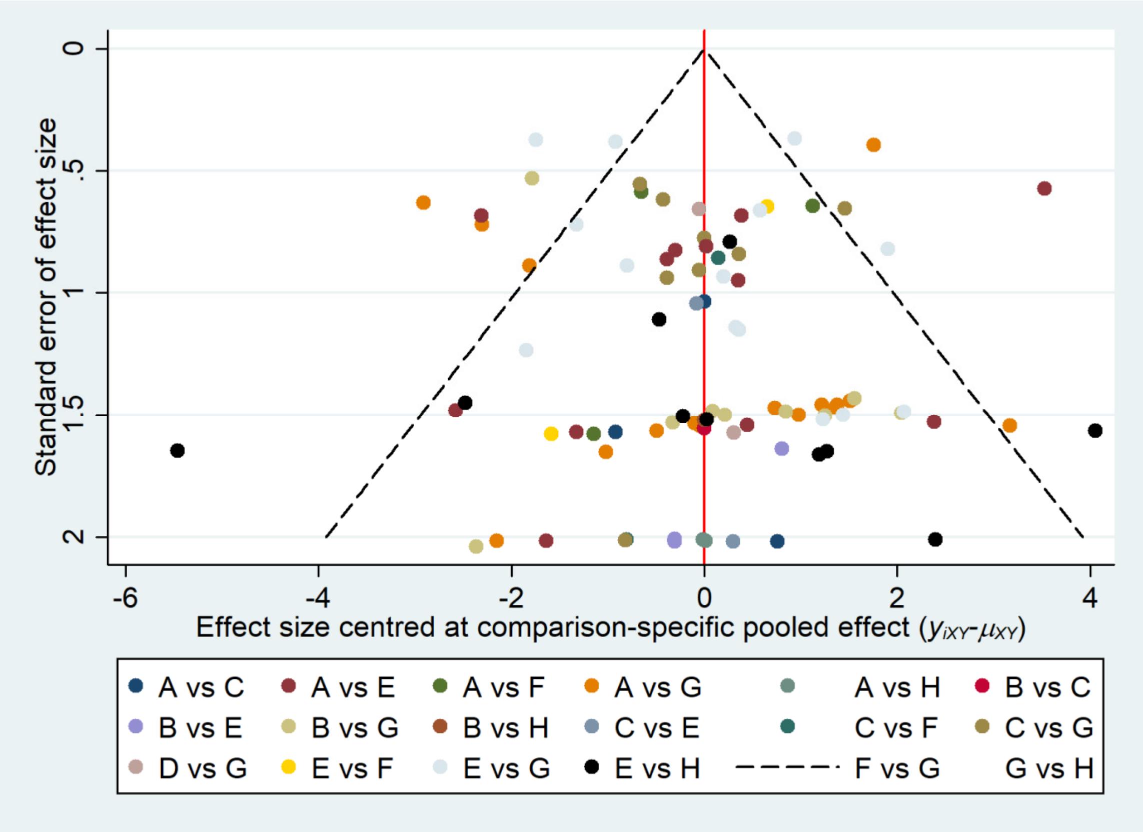

(D)

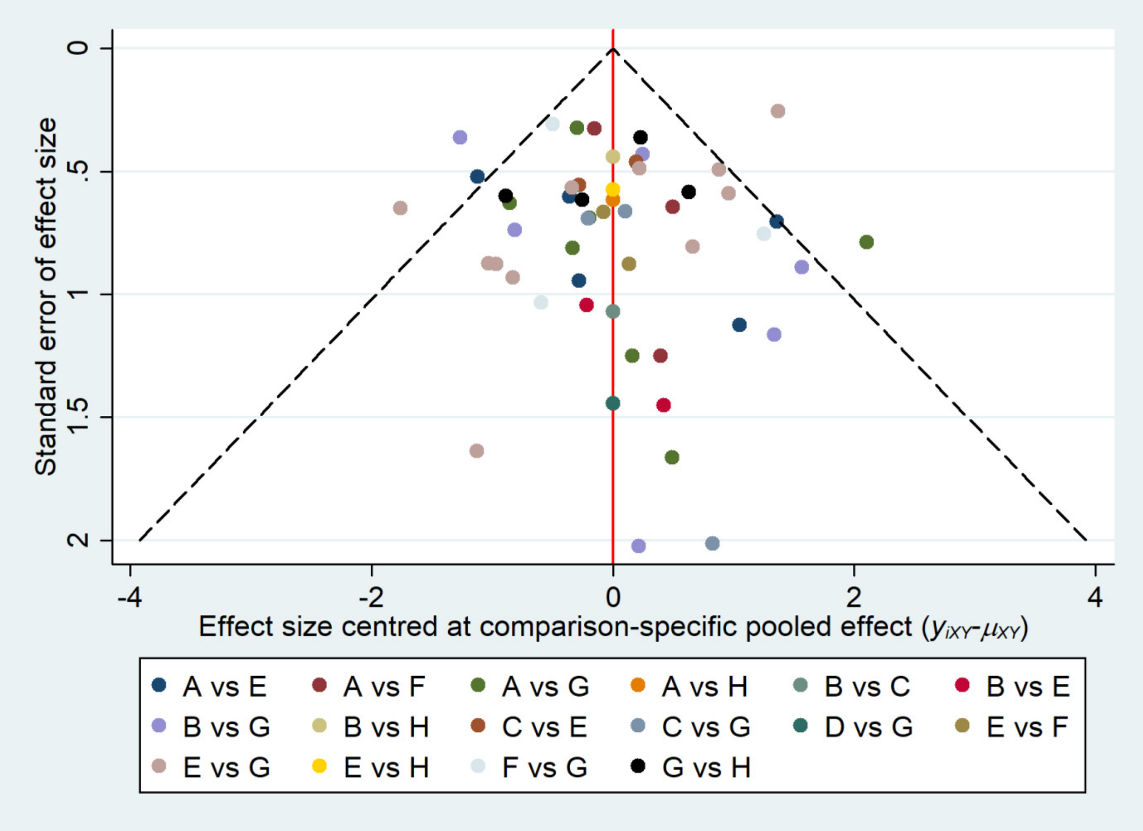

Figure S2. Comparison-adjusted funnel plot for the network meta-analysis of (A) the success at first intubation attempts, (B) intubation time, (C) glottic view and (D) sore throat in in all circumstances of airway. A, Airtraq video laryngoscope; B, Pentax Airwayscope; C, C-MAC video laryngoscope; D, C-MAC D-Blade video laryngoscope; E, GlideScope; F, King Vision video laryngoscope; G, Macintosh direct laryngoscope; H, McGrath video laryngoscope.

(A)

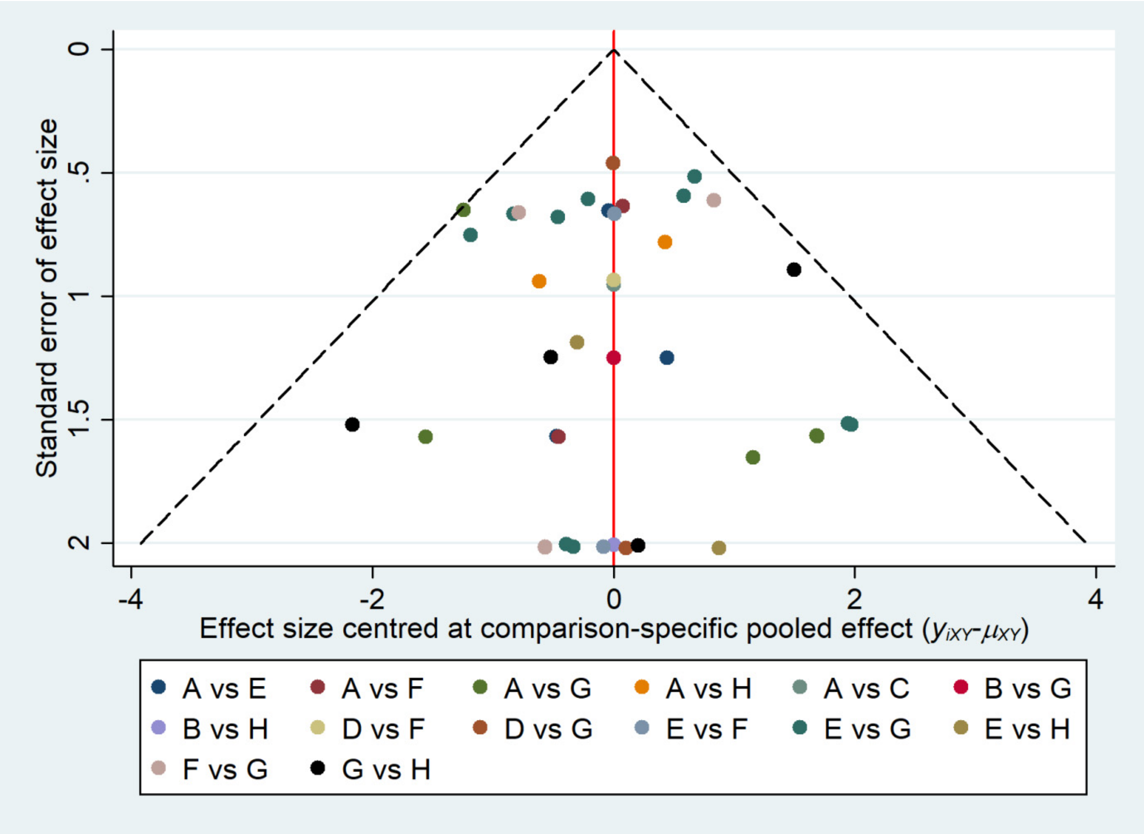

0
.5
1
1.5
2
Standard error of effect size
-4
-2
0
2
4
Effect size centred at comparison-specific pooled effect (y_{iXY}-μ_{XY})
A vs E
A vs F
A vs G
A vs H
A vs C
B vs G
B vs H
D vs F
D vs G
E vs F
E vs G
E vs H
F vs G
G vs H

(B)

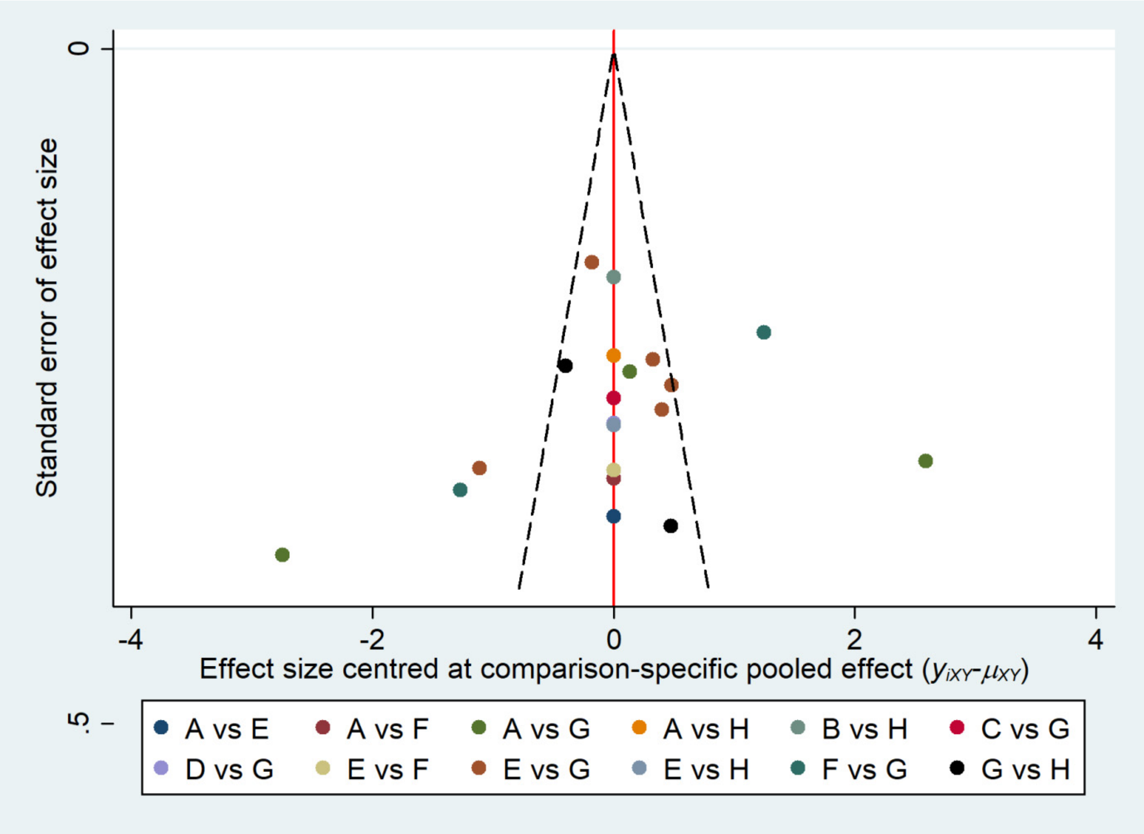

0
.5
Standard error of effect size
-4
-2
0
2
4
Effect size centred at comparison-specific pooled effect (y_{iXY}-μ_{XY})
A vs E
A vs F
A vs G
A vs H
B vs H
C vs G
D vs G
E vs F
E vs G
E vs H
F vs G
G vs H

(C)

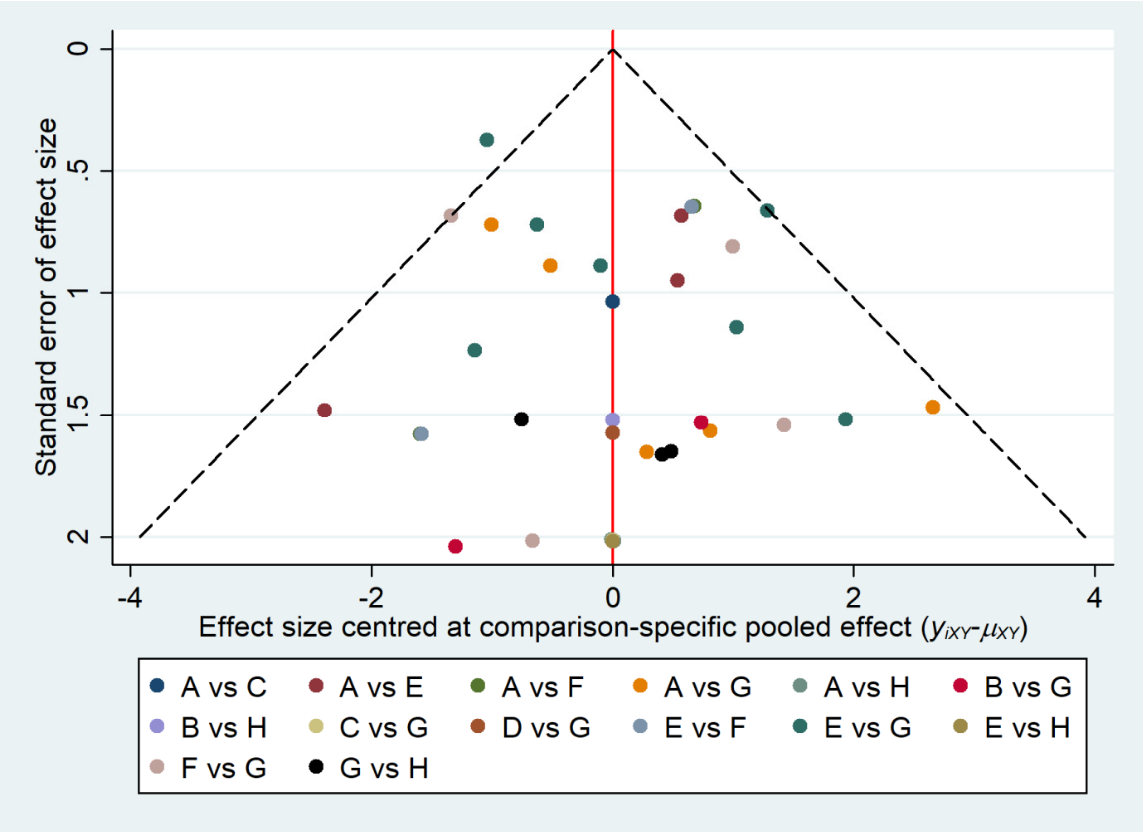

(D)

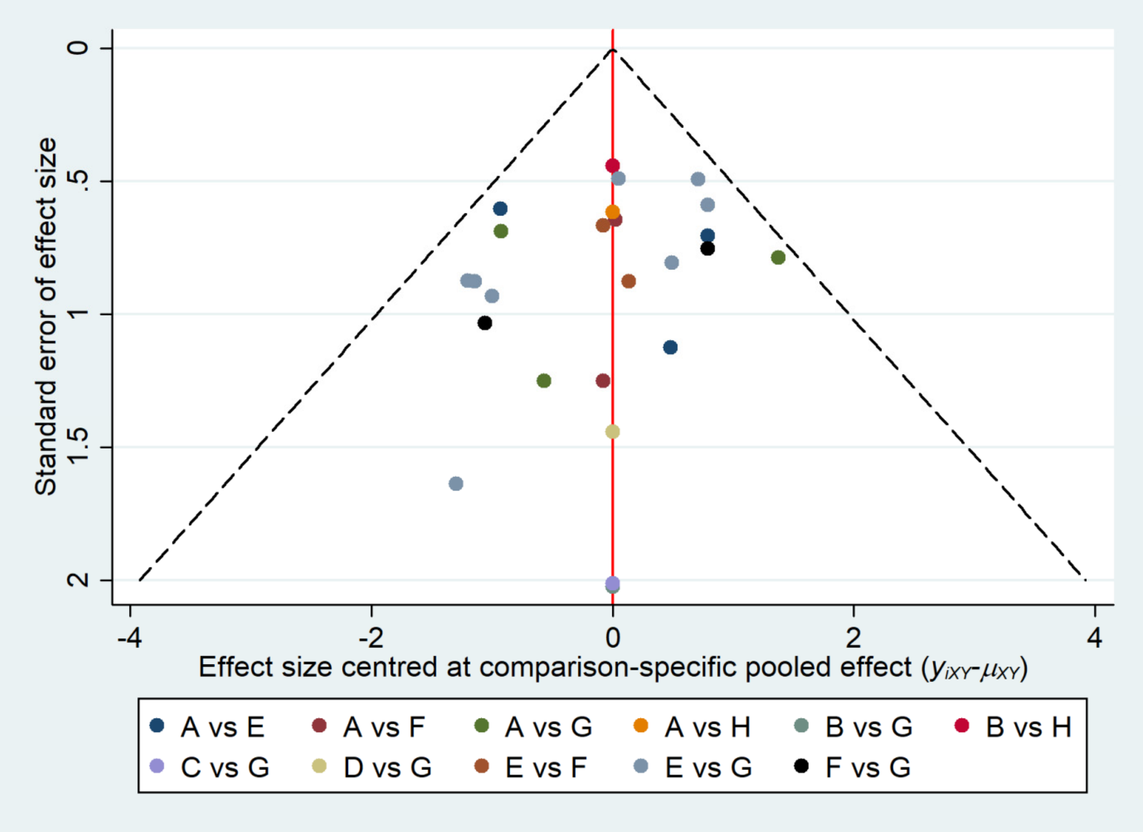

Figure S3. Comparison-adjusted funnel plot for the network meta-analysis of (A) the success at first intubation attempts, (B) intubation time, (C) glottic view and (D) sore throat in in normal airway. A, Airtraq video laryngoscope; B, Pentax Airwayscope; C, C-MAC video laryngoscope; D, C-MAC D-Blade video laryngoscope; E, GlideScope; F, King Vision video laryngoscope; G, Macintosh direct laryngoscope; H, McGrath video laryngoscope.

(A)

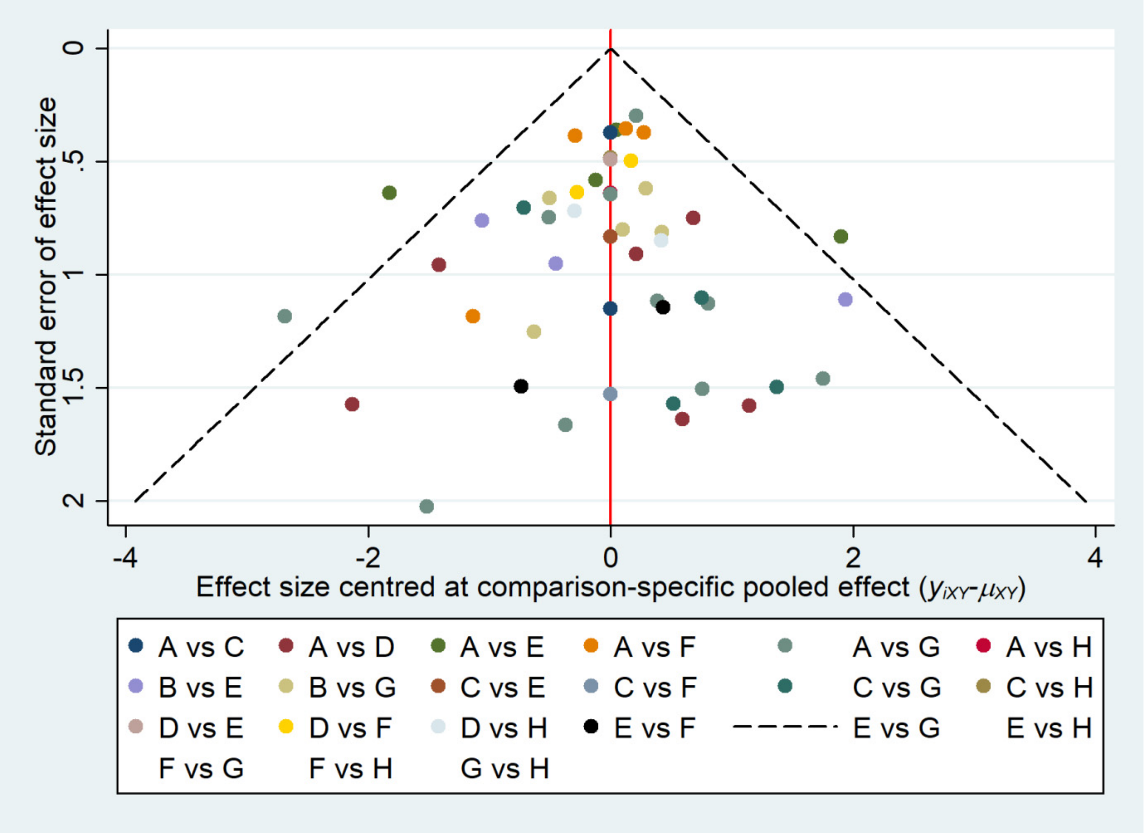
Standard error of effect size
0
.5
1
1.5
2
-4
-2
0
2
4
Effect size centred at comparison-specific pooled effect ($y_{iXY}-\mu_{XY}$)
A vs C
A vs D
A vs E
A vs F
A vs G
A vs H
B vs E
B vs G
C vs E
C vs F
C vs G
C vs H
D vs E
D vs F
D vs H
E vs F
E vs G
E vs H
F vs G
F vs H
G vs H

(B)

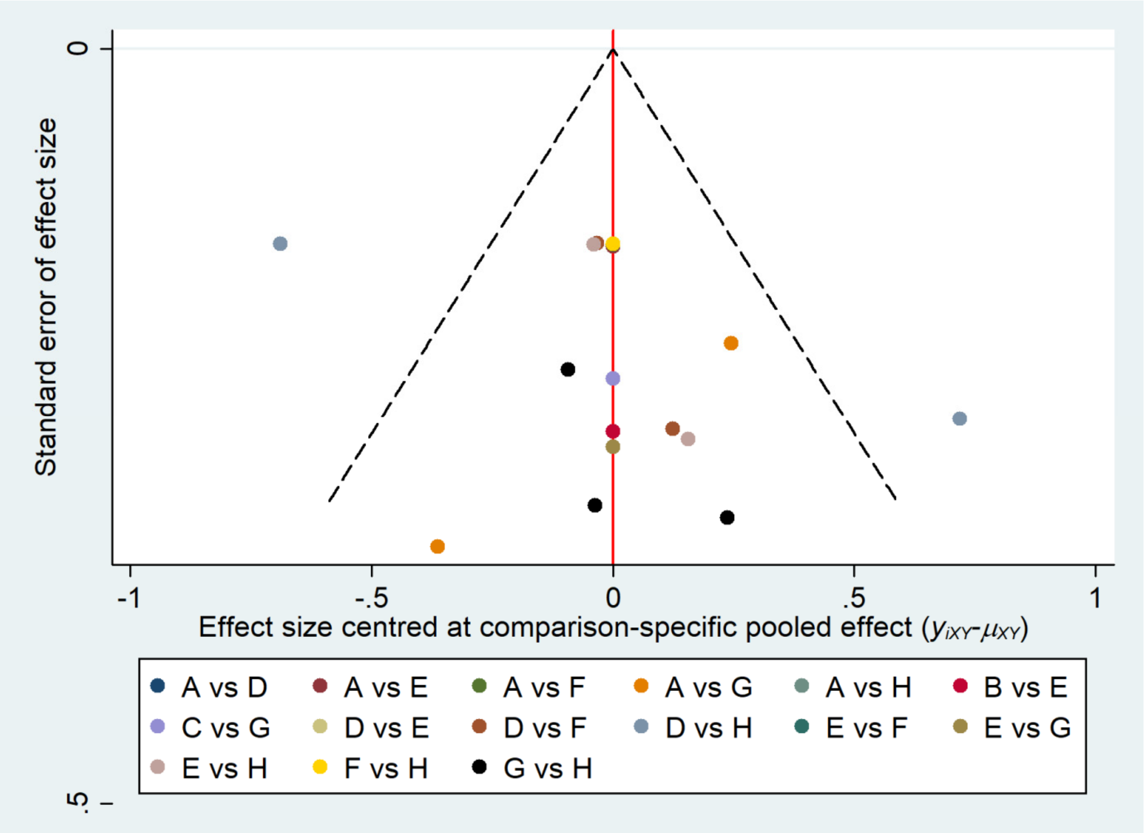
Standard error of effect size
0
.5
-1
-.5
0
.5
1
Effect size centred at comparison-specific pooled effect ($y_{iXY}-\mu_{XY}$)
A vs D
A vs E
A vs F
A vs G
A vs H
B vs E
C vs G
D vs E
D vs F
D vs H
E vs F
E vs G
E vs H
F vs H
G vs H

(C)

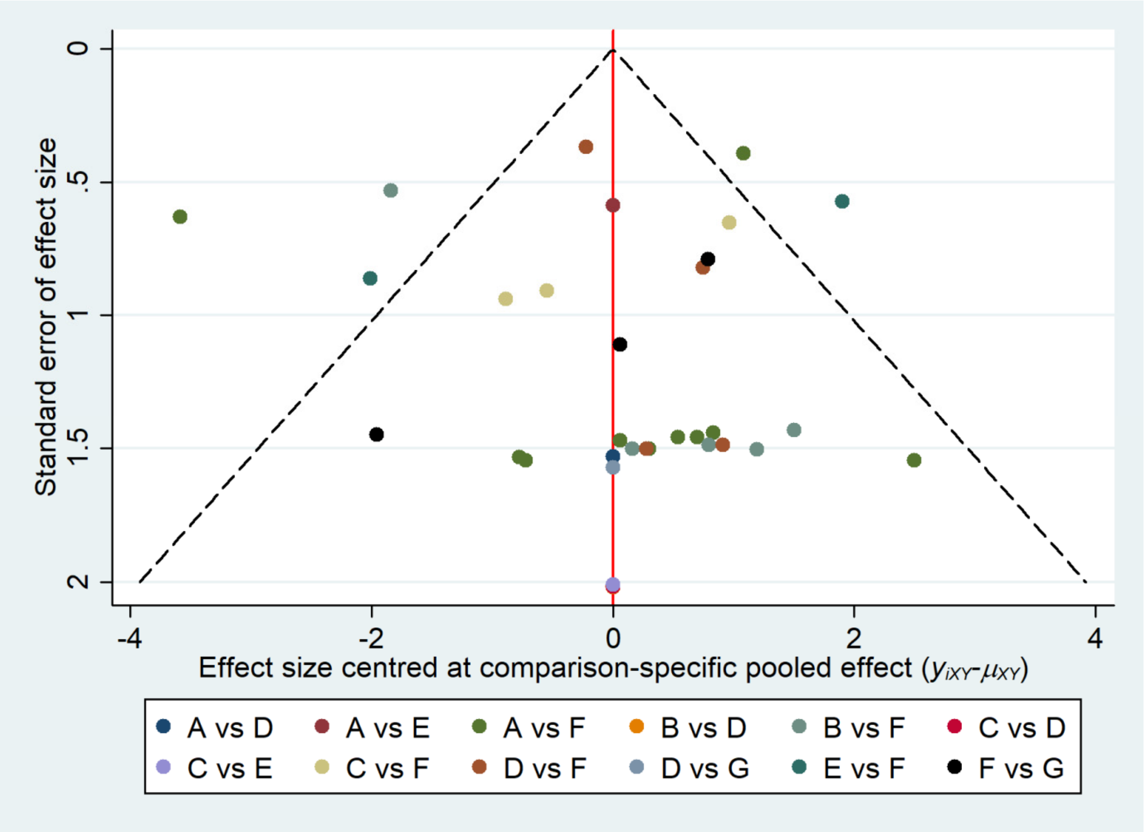

(D)

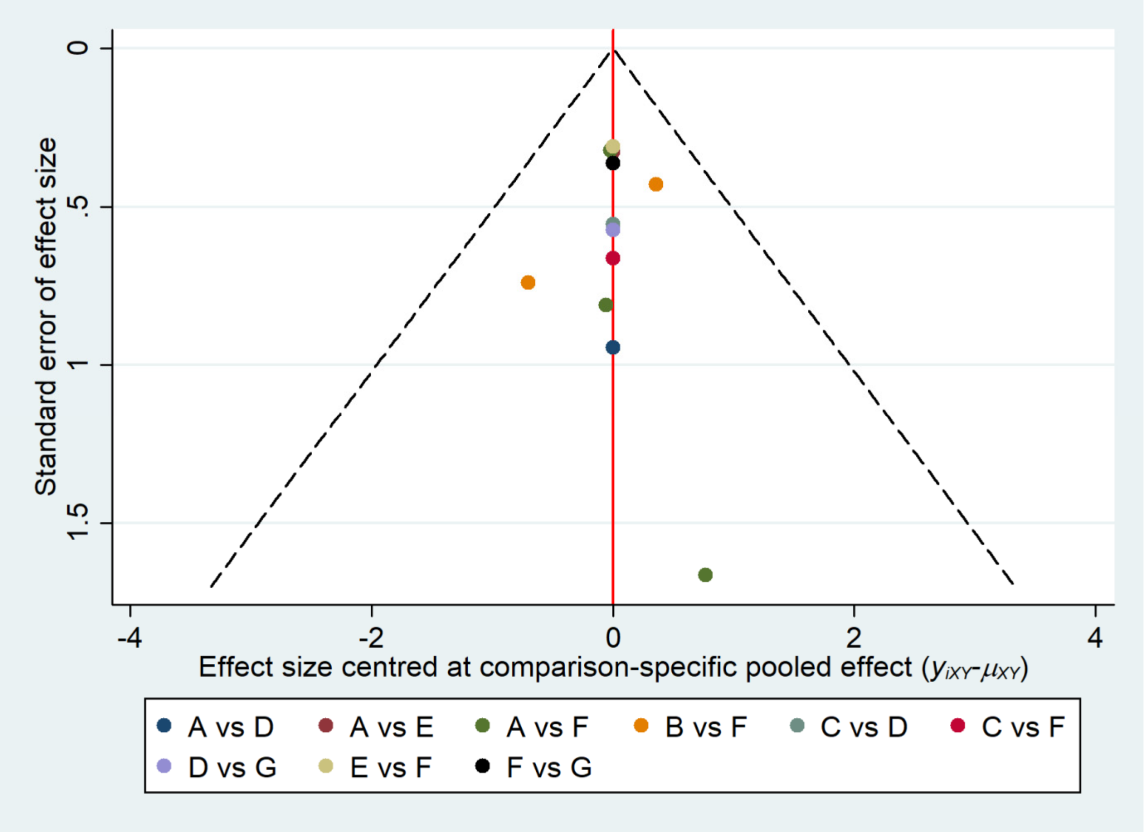

Figure S4. Comparison-adjusted funnel plot for the network meta-analysis of (A) the success at first intubation attempts, (B) intubation time, (C) glottic view and (D) sore throat in difficult airway. A, Airtraq video laryngoscope; B, Pentax Airwayscope; C, C-MAC video laryngoscope; D, C-MAC D-Blade video laryngoscope; E, GlideScope; F, King Vision video laryngoscope; G, Macintosh direct laryngoscope; H, McGrath video laryngoscope. In (C) and (D), D, GlideScope; E, King Vision video laryngoscope; F, Macintosh direct laryngoscope; G, McGrath video laryngoscope.
