# Supplementary material for: Comparisons of Videolaryngoscopes for Intubation Undergoing General Anesthesia: Systematic Review and Network Meta-Analysis of Randomized Controlled Trials
Source: J Pers Med. 2022 Feb 26;12(3):363. doi: 10.3390/jpm12030363 (PMC8954588; doi:10.3390/jpm12030363)
Supplement: Supplementary file 1 [file jpm-12-00363-s001.zip › Table S1.pdf]

Participants (studies) Follow up	Risk of bias	Inconsistency	Indirectness	Imprecision	Publication bias	Overall certainty of evidence	Study event rates (%)		Relative effect (95% CI)	Anticipated absolute effects	
							Control	Experimental		Risk with Control	Risk difference with Experimental
**ATQ compared to CMD for Normal airway**											
**Success**	N/A										
**Intubation time**	N/A										
**Glottic view**	N/A										
**Safety**	N/A										
**ATQ compared to CMD for Difficult airway**											
**Success**											
240 (1 RCT)	serious	not serious	not serious	serious	none	⨁⨁◯◯ LOW	114/120 (95.0%)	102/120 (85.0%)	**OR 0.51** (0.13 to 2.01)	950 per 1,000	**44 fewer per 1,000** (from 238 fewer to 24 more)
**Intubation time**											
240 (1 RCT)	serious	not serious	not serious	not serious	none	⨁⨁⨁◯ MODERATE	120	120	-	-	SMD **0.58 SD lower** (0.83 lower to 0.32 lower)
**Glottic view**	N/A										
**Safety**	N/A										
**ATQ compared to CM for Normal airway**											
**Success**											

60 (1 RCT)	serious	not serious	not serious	serious	none	⊕⊕◯◯ LOW	28/30 (93.3%)	27/30 (90.0%)	**OR 0.64** (0.08 to 5.17)	933 per 1,000	**34 fewer per 1,000** (from 405 fewer to 53 more)
**Intubation time**	N/A										
**Glottic view**											
60 (1 RCT)	serious	not serious	not serious	serious	none	⊕⊕◯◯ LOW	28/30 (93.3%)	28/30 (93.3%)	**OR 1.00** (0.08 to 11.87)	933 per 1,000	**0 fewer per 1,000** (from 405 fewer to 61 more)
**Safety**	N/A										
**ATQ compared to CM for Difficult airway**											
**Success**											
59 (1 RCT)	serious	not serious	not serious	serious	none	⊕⊕◯◯ LOW	26/30 (86.7%)	28/29 (96.6%)	**OR 0.83** (0.25 to 2.77)	867 per 1,000	**23 fewer per 1,000** (from 248 fewer to 81 more)
**Intubation time**	N/A										
**Glottic view**	N/A										
**Safety**	N/A										
**ATQ compared to KV for Normal airway**											
**Success**											

110 (2 RCTs)	serious	not serious	not serious	serious	none	⊕⊕◯◯ LOW	47/54 (87.0%)	42/56 (75.0%)	**OR 0.47** (0.16 to 1.41)	870 per 1,000	**111 fewer per 1,000** (from 352 fewer to 34 more)
**Intubation time**											
43 (1 RCT)	serious	not serious	not serious	not serious	none	⊕⊕⊕◯ MODERATE	22	21	-	-	SMD **1.14 SD higher** (0.49 higher to 1.79 higher)
**Glottic view**											
110 (2 RCTs)	serious	serious	not serious	not serious	none	⊕⊕◯◯ LOW	47/54 (87.0%)	49/56 (87.5%)	**OR 1.12** (0.22 to 5.84)	870 per 1,000	**12 more per 1,000** (from 274 fewer to 105 more)
**Safety**											
110 (2 RCTs)	serious	not serious	not serious	serious	none	⊕⊕◯◯ LOW	41/54 (75.9%)	49/56 (87.5%)	**OR 1.28** (0.37 to 4.42)	759 per 1,000	**42 more per 1,000** (from 221 fewer to 174 more)
**ATQ compared to KV for Difficult airway**											
**Success**											
480 (2 RCTs)	serious	not serious	not serious	serious	none	⊕⊕◯◯ LOW	212/240 (88.3%)	200/240 (83.3%)	**OR 0.93** (0.31 to 2.79)	883 per 1,000	**8 fewer per 1,000** (from 182 fewer to 71 more)
**Intubation time**											

240 (1 RCT)	serious	not serious	not serious	not serious	none	⨁⨁⨁◯ MODERATE	120	120	-	-	SMD **0.62 SD lower** (0.88 lower to 0.37 lower)
**Glottic view**											
240 (1 RCT)	serious	not serious	not serious	serious	none	⨁⨁◯◯ LOW	116/120 (96.7%)	107/120 (89.2%)	**OR 0.28** (0.02 to 4.10)	967 per 1,000	**76 fewer per 1,000** (from 600 fewer to 25 more)
**Safety**											
240 (1 RCT)	serious	not serious	not serious	serious	none	⨁⨁◯◯ LOW	84/120 (70.0%)	99/120 (82.5%)	**OR 1.33** (0.68 to 2.57)	700 per 1,000	**56 more per 1,000** (from 87 fewer to 157 more)
**ATQ compared to GVL for Normal airway**											
**Success**											
181 (3 RCTs)	serious	not serious	not serious	serious	none	⨁⨁◯◯ LOW	83/90 (92.2%)	75/91 (82.4%)	**OR 0.56** (0.23 to 1.38)	922 per 1,000	**53 fewer per 1,000** (from 191 fewer to 20 more)
**Intubation time**											
42 (1 RCT)	serious	not serious	not serious	not serious	none	⨁⨁⨁◯ MODERATE	21	21	-	-	SMD **2.43 SD higher** (1.62 higher to 3.24 higher)
**Glottic view**											

181<br>(3 RCTs)	serious	not serious	not serious	serious	none	⊕⊕◯◯<br>LOW	83/90<br>(92.2%)	77/91 (84.6%)	**OR 1.02**<br>(0.23 to 4.56)	922 per 1,000	**1 more per 1,000**<br>(from 191 fewer to 60 more)
**Safety**											
181<br>(3 RCTs)	serious	not serious	not serious	not serious	none	⊕⊕⊕◯<br>MODERATE	61/90<br>(67.8%)	78/91 (85.7%)	**OR 2.78**<br>(1.13 to 6.81)	678 per 1,000	**176 more per 1,000**<br>(from 26 more to 257 more)
**ATQ compared to GVL for Difficult airway**											
**Success**											
312<br>(2 RCTs)	serious	not serious	not serious	serious	none	⊕⊕◯◯<br>LOW	131/156<br>(84.0%)	130/156<br>(83.3%)	**OR 1.14**<br>(0.45 to 2.91)	840 per 1,000	**17 more per 1,000**<br>(from 138 fewer to 99 more)
**Intubation time**											
240<br>(1 RCT)	serious	not serious	not serious	not serious	none	⊕⊕⊕◯<br>MODERATE	120	120	-	-	SMD **0.71 SD lower**<br>(0.97 lower to 0.45 lower)
**Glottic view**											
72<br>(1 RCT)	serious	not serious	not serious	serious	none	⊕⊕◯◯<br>LOW	33/36<br>(91.7%)	36/36<br>(100.0%)	**OR 7.63**<br>(0.16 to 358.10)	917 per 1,000	**72 more per 1,000**<br>(from 279 fewer to 83 more)
**Safety**											

72<br>(1 RCT)	serious	not serious	not serious	serious	none	⊕⊕◯◯<br>LOW	33/36<br>(91.7%)	34/36 (94.4%)	**OR 1.43**<br>(0.49 to 4.15)	917 per 1,000	**24 more per 1,000**<br>(from 73 fewer to 62 more)
**ATQ compared to MG for Normal airway**											
**Success**											
147<br>(2 RCTs)	serious	not serious	not serious	serious	none	⊕⊕◯◯<br>LOW	67/74<br>(90.5%)	67/73 (91.8%)	**OR 0.79**<br>(0.29 to 2.12)	905 per 1,000	**22 fewer per 1,000**<br>(from 170 fewer to 48 more)
**Intubation time**											
87<br>(1 RCT)	serious	not serious	not serious	not serious	none	⊕⊕⊕◯<br>MODERATE	44	43	-	-	SMD **0.97 SD lower**<br>(1.42 lower to 0.52 lower)
**Glottic view**											
147<br>(2 RCTs)	serious	not serious	not serious	serious	none	⊕⊕◯◯<br>LOW	74/74<br>(100.0%)	73/73<br>(100.0%)	**OR 0.99**<br>(0.05 to 19.17)	1,000 per 1,000	**0 fewer per 1,000**<br>(from 0 fewer to 0 fewer)
**Safety**											
87<br>(1 RCT)	serious	not serious	not serious	serious	none	⊕⊕◯◯<br>LOW	39/44<br>(88.6%)	35/43 (81.4%)	**OR 0.75**<br>(0.16 to 3.43)	886 per 1,000	**32 fewer per 1,000**<br>(from 331 fewer to 78 more)
**ATQ compared to MG for Difficult airway**											
**Success**											

240<br>(1 RCT)	serious	not serious	not serious	serious	none	⊕⊕◯◯<br>LOW	117/120<br>(97.5%)	102/120<br>(85.0%)	**OR 0.78**<br>(0.16 to 3.43)	975 per 1,000	**7 fewer per 1,000**<br>(from 113 fewer to 18 more)
**Intubation time**											
240<br>(1 RCT)	serious	not serious	not serious	not serious	none	⊕⊕⊕◯<br>MODERATE	120	120	-	-	SMD **0.43 SD lower**<br>(0.68 lower to 0.17 lower)
**Glottic view**	N/A										
**Safety**	N/A										
**ATQ compared to MCL for Normal airway**											
**Success**											
320<br>(5 RCTs)	serious	not serious	not serious	serious	none	⊕⊕◯◯<br>LOW	148/159<br>(93.1%)	147/161<br>(91.3%)	**OR 0.87**<br>(0.37 to 2.04)	931 per 1,000	**10 fewer per 1,000**<br>(from 98 fewer to 34 more)
**Intubation time**											
193<br>(3 RCTs)	serious	serious	not serious	not serious	none	⊕⊕◯◯<br>LOW	97	96	-	-	SMD **0.02 SD higher**<br>(2.58 lower to 2.62 higher)
**Glottic view**											
320<br>(5 RCTs)	serious	not serious	not serious	serious	none	⊕⊕◯◯<br>LOW	138/159<br>(86.8%)	154/161<br>(95.7%)	**OR 8.07**<br>(0.70 to 93.52)	868 per 1,000	**114 more per 1,000**<br>(from 46 fewer to 130 more)

**Safety**											
190 (3 RCTs)	serious	not serious	not serious	not serious	none	⊕⊕⊕◯ MODERATE	37/94 (39.4%)	55/96 (57.3%)	**OR 3.13** (1.26 to 7.80)	394 per 1,000	**277 more per 1,000** (from 56 more to 441 more)
**ATQ compared to MCL for Difficult airway**											
**Success**											
778 (9 RCTs)	serious	not serious	not serious	not serious	none	⊕⊕⊕◯ MODERATE	285/388 (73.5%)	360/390 (92.3%)	**OR 3.06** (1.39 to 6.72)	735 per 1,000	**160 more per 1,000** (from 59 more to 214 more)
**Intubation time**											
172 (2 RCTs)	serious	serious	not serious	not serious	none	⊕⊕◯◯ LOW	84	88	-	-	SMD **1.17 SD lower** (1.75 lower to 0.58 lower)
**Glottic view**											
1020 (10 RCTs)	serious	not serious	not serious	serious	none	⊕⊕◯◯ LOW	324/508 (63.8%)	490/512 (95.7%)	**OR 0.24** (0.01 to 3.94)	638 per 1,000	**341 fewer per 1,000** (from 620 fewer to 236 more)
**Safety**											
545 (3 RCTs)	serious	not serious	not serious	serious	none	⊕⊕◯◯ LOW	183/266 (68.8%)	234/279 (83.9%)	**OR 1.48** (0.82 to 2.68)	688 per 1,000	**77 more per 1,000** (from 44 fewer to 167 more)
**AWS compared to CM for Normal airway**											
**Success**	N/A										

**Intubation time**	N/A										
**Glottic view**	N/A										
**Safety**	N/A										
**AWS compared to CM for Difficult airway**											
**Success**	N/A										
**Intubation time**	N/A										
**Glottic view**	N/A										
**Safety**	N/A										
**AWS compared to MCL for Normal airway**											
**Success**											
68 (1 RCT)	serious	not serious	not serious	serious	none	⊕⊕◯◯ LOW	32/34 (94.1%)	31/34 (91.2%)	**OR 1.71** (0.19 to 15.72)	941 per 1,000	**24 more per 1,000** (from 189 fewer to 55 more)
**Intubation time**	N/A										
**Glottic view**											
92 (2 RCTs)	serious	not serious	not serious	serious	none	⊕⊕◯◯ LOW	43/46 (93.5%)	46/46 (100.0%)	**OR 1.09** (0.25 to 4.86)	935 per 1,000	**5 more per 1,000** (from 153 fewer to 51 more)
**Safety**											

40<br>(1 RCT)	serious	not serious	not serious	serious	none	⊕⊕◯◯<br>LOW	20/20<br>(100.0%)	20/20<br>(100.0%)	**OR 6.54**<br>(0.83 to 51.70)	1,000 per 1,000	**0 fewer per 1,000**<br>(from 0 fewer to 0 fewer)
**AWS compared to MCL for Difficult airway**											
**Success**											
305<br>(5 RCTs)	serious	not serious	not serious	serious	none	⊕⊕◯◯<br>LOW	131/152<br>(86.2%)	130/153<br>(85.0%)	**OR 1.29**<br>(0.49 to 3.38)	862 per 1,000	**28 more per 1,000**<br>(from 108 fewer to 93 more)
**Intubation time**	N/A										
**Glottic view**											
711<br>(6 RCTs)	serious	not serious	not serious	not serious	none	⊕⊕⊕◯<br>MODERATE	295/355<br>(83.1%)	349/356<br>(98.0%)	**OR 49.84**<br>(3.97 to 626.44)	831 per 1,000	**165 more per 1,000**<br>(from 120 more to 169 more)
**Safety**											
135<br>(2 RCTs)	serious	not serious	not serious	serious	none	⊕⊕◯◯<br>LOW	47/67<br>(70.1%)	44/68 (64.7%)	**OR 0.78**<br>(0.35 to 1.76)	701 per 1,000	**54 fewer per 1,000**<br>(from 250 fewer to 104 more)
**AWS compared to GVL for Normal airway**											
**Success**	N/A										
**Intubation time**	N/A										
**Glottic view**	N/A										

**Safety**	N/A										
**AWS compared to GVL for Difficult airway**											
**Success**											
180 (3 RCTs)	serious	not serious	not serious	serious	none	⨁⨁◯◯ LOW	79/90 (87.8%)	79/90 (87.8%)	**OR 0.48** (0.16 to 1.42)	878 per 1,000	**103 fewer per 1,000** (from 343 fewer to 33 more)
**Intubation time**											
70 (1 RCT)	serious	not serious	not serious	not serious	none	⨁⨁⨁◯ MODERATE	35	35	-	-	SMD **0.97 SD lower** (1.47 lower to 0.48 lower)
**Glottic view**											
180 (3 RCTs)	serious	not serious	not serious	serious	none	⨁⨁◯◯ LOW	90/90 (100.0%)	90/90 (100.0%)	**OR 1.00** (0.01 to 102.05)	1,000 per 1,000	**0 fewer per 1,000** (from 0 fewer to 0 fewer)
**Safety**	N/A										
**AWS compared to MG for Normal airway**											
**Success**											
140 (1 RCT)	serious	not serious	not serious	serious	none	⨁⨁◯◯ LOW	70/70 (100.0%)	70/70 (100.0%)	**OR 1.55** (0.16 to 15.56)	1,000 per 1,000	**0 fewer per 1,000** (from 0 fewer to 0 fewer)
**Intubation time**											

140 (1 RCT)	serious	not serious	not serious	serious	none	⊕⊕◯◯ LOW	70	70	-	-	SMD **0.17 SD lower** (0.51 lower to 0.16 higher)
**Glottic view**											
140 (1 RCT)	serious	not serious	not serious	serious	none	⊕⊕◯◯ LOW	67/70 (95.7%)	70/70 (100.0%)	**OR 3.20** (0.05 to 218.49)	957 per 1,000	**29 more per 1,000** (from 430 fewer to 43 more)
**Safety**											
140 (1 RCT)	serious	not serious	not serious	serious	none	⊕⊕◯◯ LOW	53/70 (75.7%)	60/70 (85.7%)	**OR 1.56** (0.41 to 5.89)	757 per 1,000	**72 more per 1,000** (from 196 fewer to 191 more)
**AWS compared to MG for Difficult airway**											
**Success**	N/A										
**Intubation time**	N/A										
**Glottic view**	N/A										
**Safety**	N/A										
**CM compared to GVL for Normal airway**											
**Success**	N/A										
**Intubation time**	N/A										
**Glottic view**	N/A										
**Safety**	N/A										

**CM compared to GVL for Difficult airway**											
**Success**											
56 (1 RCT)	serious	not serious	not serious	serious	none	⊕⊕◯◯ LOW	28/30 (93.3%)	15/26 (57.7%)	**OR 1.38** (0.44 to 4.35)	933 per 1,000	**17 more per 1,000** (from 73 fewer to 51 more)
**Intubation time**	N/A										
**Glottic view**											
56 (1 RCT)	serious	not serious	not serious	serious	none	⊕⊕◯◯ LOW	30/30 (100.0%)	26/26 (100.0%)	**OR 0.87** (0.01 to 89.19)	1,000 per 1,000	**0 fewer per 1,000** (from 0 fewer to 0 fewer)
**Safety**											
56 (1 RCT)	serious	not serious	not serious	serious	none	⊕⊕◯◯ LOW	18/30 (60.0%)	17/26 (65.4%)	**OR 0.83** (0.33 to 2.13)	600 per 1,000	**45 fewer per 1,000** (from 269 fewer to 162 more)
**CM compared to MG for Normal airway**											
**Success**	N/A										
**Intubation time**	N/A										
**Glottic view**	N/A										
**Safety**	N/A										
**CM compared to MG for Difficult airway**											
**Success**											

130 (1 RCT)	serious	not serious	not serious	serious	none	⨁⨁◯◯ LOW	45/65 (69.2%)	58/65 (89.2%)	**OR 0.94** (0.27 to 3.27)	692 per 1,000	**13 fewer per 1,000** (from 314 fewer to 188 more)
**Intubation time**	N/A										
**Glottic view**	N/A										
**Safety**	N/A										
**CM compared to KV for Normal airway**											
**Success**	N/A										
**Intubation time**	N/A										
**Glottic view**	N/A										
**Safety**	N/A										
**CM compared to KV for Difficult airway**											
**Success**											
90 (1 RCT)	serious	not serious	not serious	serious	none	⨁⨁◯◯ LOW	42/45 (93.3%)	45/45 (100.0%)	**RR 1.11** (0.29 to 4.25)	933 per 1,000	**103 more per 1,000** (from 663 fewer to 1,000 more)
**Intubation time**	N/A										
**Glottic view**											

90<br>(1 RCT)	serious	not serious	not serious	serious	none	⨁⨁◯◯<br>LOW	45/45<br>(100.0%)	45/45<br>(100.0%)	**OR 0.03**<br>(0.00 to 23.44)	1,000 per 1,000	**0 fewer per 1,000**<br>(from 0 fewer to --)
**Safety**	N/A										
**CM compared to MCL for Normal airway**											
**Success**	N/A										
**Intubation time**											
78<br>(1 RCT)	serious	not serious	not serious	not serious	none	⨁⨁⨁◯<br>MODERATE	39	39	-	-	SMD **1.53 SD lower**<br>(2.04 lower to 1.02 lower)
**Glottic view**											
78<br>(1 RCT)	serious	not serious	not serious	serious	none	⨁⨁◯◯<br>LOW	39/39<br>(100.0%)	39/39<br>(100.0%)	**OR 2.29**<br>(0.01 to 487.52)	1,000 per 1,000	**0 fewer per 1,000**<br>(from 0 fewer to 0 fewer)
**Safety**											
78<br>(1 RCT)	serious	not serious	not serious	serious	none	⨁⨁◯◯<br>LOW	39/39<br>(100.0%)	39/39<br>(100.0%)	**OR 1.00**<br>(0.02 to 59.45)	1,000 per 1,000	**0 fewer per 1,000**<br>(from 0 fewer to 0 fewer)
**CM compared to MCL for Difficult airway**											
**Success**											
289<br>(4 RCTs)	serious	not serious	not serious	serious	none	⨁⨁◯◯<br>LOW	126/145<br>(86.9%)	139/144<br>(96.5%)	**OR 1.11**<br>(0.29 to 4.25)	869 per 1,000	**11 more per 1,000**<br>(from 211 fewer to 97 more)

**Intubation time**											
90 (1 RCT)	serious	not serious	not serious	not serious	none	⊕⊕⊕◯ MODERATE	45	45	-	-	SMD **0.76 SD lower** (1.19 lower to 0.33 lower)
**Glottic view**											
210 (3 RCTs)	serious	not serious	not serious	serious	none	⊕⊕◯◯ LOW	80/105 (76.2%)	97/105 (92.4%)	**OR 2.77** (0.00 to 3219.78)	762 per 1,000	**137 more per 1,000** (from -- to 238 more)
**Safety**											
75 (1 RCT)	serious	not serious	not serious	serious	none	⊕⊕◯◯ LOW	32/36 (88.9%)	31/39 (79.5%)	**OR 0.86** (0.31 to 2.44)	889 per 1,000	**16 fewer per 1,000** (from 176 fewer to 62 more)
**KV compared to MG for Normal airway**											
**Success**	N/A										
**Intubation time**	N/A										
**Glottic view**	N/A										
**Safety**	N/A										
**KV compared to MG for Difficult airway**											
**Success**											
240 (1 RCT)	serious	not serious	not serious	serious	none	⊕⊕◯◯ LOW	117/120 (97.5%)	104/120 (86.7%)	**OR 0.84** (0.23 to 3.08)	975 per 1,000	**5 fewer per 1,000** (from 75 fewer to 17 more)

**Intubation time**											
240<br>(1 RCT)	serious	not serious	not serious	serious	none	⊕⊕◯◯<br>LOW	120	120	-	-	SMD **0.15 SD higher**<br>(0.11 lower to 0.4 higher)
**Glottic view**	N/A										
**Safety**	N/A										
**KV compared to MCL for Normal airway**											
**Success**											
266<br>(4 RCTs)	serious	not serious	not serious	serious	none	⊕⊕◯◯<br>LOW	114/133<br>(85.7%)	122/133<br>(91.7%)	**OR 1.83**<br>(0.76 to 4.40)	857 per 1,000	**59 more per 1,000**<br>(from 37 fewer to 106 more)
**Intubation time**											
144<br>(2 RCTs)	serious	serious	not serious	not serious	none	⊕⊕◯◯<br>LOW	72	72	-	-	SMD **0.22 SD higher**<br>(2.03 lower to 2.47 higher)
**Glottic view**											
266<br>(4 RCTs)	serious	not serious	not serious	serious	none	⊕⊕◯◯<br>LOW	117/133<br>(88.0%)	124/133<br>(93.2%)	**OR 1.09**<br>(0.06 to 19.66)	880 per 1,000	**9 more per 1,000**<br>(from 575 fewer to 113 more)
**Safety**											

108 (2 RCTs)	serious	serious	not serious	not serious	none	⨁⨁◯◯ LOW	33/54 (61.1%)	41/54 (75.9%)	**OR 2.44** (0.75 to 7.95)	611 per 1,000	**182 more per 1,000** (from 70 fewer to 315 more)
**KV compared to MCL for Difficult airway**											
**Success**											
300 (2 RCTs)	serious	not serious	not serious	not serious	none	⨁⨁⨁◯ MODERATE	80/150 (53.3%)	137/150 (91.3%)	**OR 3.31** (1.14 to 9.56)	533 per 1,000	**258 more per 1,000** (from 32 more to 383 more)
**Intubation time**	N/A										
**Glottic view**											
300 (2 RCTs)	serious	not serious	not serious	serious	none	⨁⨁◯◯ LOW	41/150 (27.3%)	144/150 (96.0%)	**OR 6.53** (0.01 to 4461.53)	273 per 1,000	**437 more per 1,000** (from 270 fewer to 726 more)
**Safety**											
240 (1 RCT)	serious	not serious	not serious	serious	none	⨁⨁◯◯ LOW	92/120 (76.7%)	94/120 (78.3%)	**OR 1.12** (0.59 to 2.21)	767 per 1,000	**20 more per 1,000** (from 107 fewer to 112 more)
**CMD compared to MG for Normal airway**											
**Success**	N/A										
**Intubation time**	N/A										
**Glottic view**	N/A										

**Safety**	N/A										
**CMD compared to MG for Difficult airway**											
**Success**											
320 (2 RCTs)	serious	not serious	not serious	serious	none	⨁⨁◯◯ LOW	154/160 (96.3%)	151/160 (94.4%)	**OR 1.54** (0.39 to 6.13)	963 per 1,000	**13 more per 1,000** (from 53 fewer to 31 more)
**Intubation time**											
320 (2 RCTs)	serious	serious	not serious	not serious	none	⨁⨁◯◯ LOW	160	160	-	-	SMD **0.52 SD lower** (1.9 lower to 0.86 higher)
**Glottic view**	N/A										
**Safety**	N/A										
**CMD compared to KV for Normal airway**											
**Success**											
100 (1 RCT)	serious	not serious	not serious	serious	none	⨁⨁◯◯ LOW	47/50 (94.0%)	48/50 (96.0%)	**OR 0.67** (0.19 to 2.34)	940 per 1,000	**27 fewer per 1,000** (from 191 fewer to 33 more)
**Intubation time**	N/A										
**Glottic view**	N/A										
**Safety**	N/A										
**CMD compared to KV for Difficult airway**											
**Success**											

303<br>(2 RCTs)	serious	not serious	not serious	serious	none	⊕⊕◯◯<br>LOW	127/151<br>(84.1%)	141/152<br>(92.8%)	**OR 1.83**<br>(0.50 to 6.69)	841 per 1,000	**65 more per 1,000**<br>(from 115 fewer to 131 more)
**Intubation time**											
303<br>(2 RCTs)	serious	not serious	not serious	serious	none	⊕⊕◯◯<br>LOW	151	152	-	-	SMD **0 SD**<br>(0.22 lower to 0.23 higher)
**Glottic view**	N/A										
**Safety**	N/A										
**CMD compared to GVL for Normal airway**											
**Success**	N/A										
**Intubation time**	N/A										
**Glottic view**	N/A										
**Safety**	N/A										
**CMD compared to GVL for Difficult airway**											
**Success**											
240<br>(1 RCT)	serious	not serious	not serious	serious	none	⊕⊕◯◯<br>LOW	102/120<br>(85.0%)	114/120<br>(95.0%)	**OR 2.26**<br>(0.57 to 8.92)	850 per 1,000	**78 more per 1,000**<br>(from 86 fewer to 131 more)
**Intubation time**											

240<br>(1 RCT)	serious	not serious	not serious	serious	none	⊕⊕◯◯<br>LOW	120	120	-	-	SMD **0.2 SD lower**<br>(0.45 lower to 0.06 higher)
**Glottic view**	N/A										
**Safety**	N/A										
**CMD compared to MCL for Normal airway**											
**Success**											
182<br>(2 RCTs)	serious	not serious	not serious	serious	none	⊕⊕◯◯<br>LOW	80/91<br>(87.9%)	79/91 (86.8%)	**OR 1.22**<br>(0.41 to 3.65)	879 per 1,000	**20 more per 1,000**<br>(from 130 fewer to 85 more)
**Intubation time**											
52<br>(1 RCT)	serious	not serious	not serious	serious	none	⊕⊕◯◯<br>LOW	26	26	-	-	SMD **0.04 SD lower**<br>(0.59 lower to 0.5 higher)
**Glottic view**											
52<br>(1 RCT)	serious	not serious	not serious	serious	none	⊕⊕◯◯<br>LOW	24/26<br>(92.3%)	26/26<br>(100.0%)	**OR 3.54**<br>(0.26 to 48.31)	923 per 1,000	**54 more per 1,000**<br>(from 166 fewer to 75 more)
**Safety**											
130<br>(1 RCT)	serious	not serious	not serious	not serious	none	⊕⊕⊕◯<br>MODERATE	21/65<br>(32.3%)	65/65<br>(100.0%)	**OR 271.14**<br>(13.20 to 5568.91)	323 per 1,000	**669 more per 1,000**<br>(from 540 more to 677 more)

**CMD compared to MCL for Difficult airway**											
**Success**	N/A										
**Intubation time**	N/A										
**Glottic view**	N/A										
**Safety**	N/A										
**GVL compared to MG for Normal airway**											
**Success**											
106 (2 RCTs)	serious	not serious	not serious	serious	none	⊕⊕◯◯ LOW	52/53 (98.1%)	50/53 (94.3%)	**OR 1.41** (0.50 to 3.97)	981 per 1,000	**5 more per 1,000** (from 18 fewer to 14 more)
**Intubation time**											
56 (1 RCT)	serious	not serious	not serious	not serious	none	⊕⊕⊕◯ MODERATE	28	28	-	-	SMD **0.81 SD lower** (1.36 lower to 0.27 lower)
**Glottic view**											
106 (2 RCTs)	serious	not serious	not serious	serious	none	⊕⊕◯◯ LOW	53/53 (100.0%)	53/53 (100.0%)	**OR 0.97** (0.03 to 26.85)	1,000 per 1,000	**0 fewer per 1,000** (from 0 fewer to 0 fewer)
**Safety**	N/A										
**GVL compared to MG for Difficult airway**											
**Success**											

300<br>(2 RCTs)	serious	not serious	not serious	serious	none	⊕⊕◯◯<br>LOW	138/150<br>(92.0%)	130/150<br>(86.7%)	**OR 0.68**<br>(0.22 to 2.11)	920 per<br>1,000	**33 fewer per 1,000**<br>(from 203 fewer to 40 more)
**Intubation time**											
300<br>(2 RCTs)	serious	not serious	not serious	not serious	none	⊕⊕⊕◯<br>MODERATE	150	150	-	-	SMD **0.32 SD higher**<br>(0.1 higher to 0.55 higher)
**Glottic view**											
60<br>(1 RCT)	serious	not serious	not serious	serious	none	⊕⊕◯◯<br>LOW	30/30<br>(100.0%)	28/30 (93.3%)	**OR 0.19**<br>(0.00 to 9.33)	1,000 per<br>1,000	**0 fewer per 1,000**<br>(from 0 fewer to --)
**Safety**											
60<br>(1 RCT)	serious	not serious	not serious	serious	none	⊕⊕◯◯<br>LOW	19/30<br>(63.3%)	23/30 (76.7%)	**OR 1.19**<br>(0.47 to 2.98)	633 per<br>1,000	**39 more per 1,000**<br>(from 185 fewer to 204 more)
**MCL compared to MG for Normal airway**											
**Success**											
263<br>(4 RCTs)	serious	serious	not serious	not serious	none	⊕⊕◯◯<br>LOW	125/132<br>(94.7%)	123/131<br>(93.9%)	**OR 0.91**<br>(0.34 to 2.39)	947 per<br>1,000	**5 fewer per 1,000**<br>(from 88 fewer to 30 more)
**Intubation time**											

113 (2 RCTs)	serious	not serious	not serious	serious	none	⊕⊕◯◯ LOW	57	56	-	-	SMD **0.79 SD lower** (1.64 lower to 0.06 higher)
**Glottic view**											
163 (3 RCTs)	serious	not serious	not serious	serious	none	⊕⊕◯◯ LOW	82/82 (100.0%)	75/81 (92.6%)	**OR 0.18** (0.01 to 5.51)	1,000 per 1,000	**0 fewer per 1,000** (from 0 fewer to 0 fewer)
**Safety**	N/A										
**MCL compared to MG for Difficult airway**											
**Success**											
93 (2 RCTs)	serious	not serious	not serious	not serious	none	⊕⊕⊕◯ MODERATE	45/46 (97.8%)	35/47 (74.5%)	**OR 0.25** (0.08 to 0.76)	978 per 1,000	**60 fewer per 1,000** (from 196 fewer to 7 fewer)
**Intubation time**											
193 (3 RCTs)	serious	not serious	not serious	not serious	none	⊕⊕⊕◯ MODERATE	96	97	-	-	SMD **0.92 SD higher** (0.62 higher to 1.22 higher)
**Glottic view**											
242 (3 RCTs)	serious	not serious	not serious	serious	none	⊕⊕◯◯ LOW	118/121 (97.5%)	70/121 (57.9%)	**OR 0.03** (0.00 to 100.82)	975 per 1,000	**434 fewer per 1,000** (from -- to 25 more)
**Safety**											

128<br>(1 RCT)	serious	not serious	not serious	serious	none	⊕⊕◯◯<br>LOW	40/64<br>(62.5%)	39/64 (60.9%)	**OR 1.14**<br>(0.54 to 2.45)	625 per 1,000	**30 more per 1,000**<br>(from 151 fewer to 178 more)
**GVL compared to KV for Normal airway**											
**Success**											
109<br>(2 RCTs)	serious	not serious	not serious	serious	none	⊕⊕◯◯<br>LOW	47/54<br>(87.0%)	49/55 (89.1%)	**OR 0.85**<br>(0.32 to 2.25)	870 per 1,000	**19 fewer per 1,000**<br>(from 188 fewer to 68 more)
**Intubation time**											
43<br>(1 RCT)	serious	not serious	not serious	not serious	none	⊕⊕⊕◯<br>MODERATE	22	21	-	-	SMD **0.82 SD lower**<br>(1.44 lower to 0.19 lower)
**Glottic view**											
109<br>(2 RCTs)	serious	not serious	not serious	serious	none	⊕⊕◯◯<br>LOW	47/54<br>(87.0%)	48/55 (87.3%)	**OR 1.10**<br>(0.21 to 5.73)	870 per 1,000	**10 more per 1,000**<br>(from 285 fewer to 104 more)
**Safety**											
109<br>(2 RCTs)	serious	not serious	not serious	serious	none	⊕⊕◯◯<br>LOW	41/54<br>(75.9%)	34/55 (61.8%)	**OR 0.46**<br>(0.15 to 1.46)	759 per 1,000	**167 fewer per 1,000**<br>(from 438 fewer to 62 more)
**GVL compared to KV for Difficult airway**											
**Success**											

240 (1 RCT)	serious	not serious	not serious	serious	none	⊕⊕◯◯ LOW	104/120 (86.7%)	102/120 (85.0%)	**OR 0.81** (0.26 to 2.53)	867 per 1,000	**26 fewer per 1,000** (from 238 fewer to 76 more)
**Intubation time**											
240 (1 RCT)	serious	not serious	not serious	serious	none	⊕⊕◯◯ LOW	120	120	-	-	SMD **0.24 SD higher** (0.01 lower to 0.5 higher)
**Glottic view**	N/A										
**Safety**	N/A										
**GVL compared to MCL for Normal airway**											
**Success**											
944 (10 RCTs)	serious	serious	not serious	not serious	none	⊕⊕◯◯ LOW	420/474 (88.6%)	440/470 (93.6%)	**OR 1.55** (0.88 to 2.73)	886 per 1,000	**37 more per 1,000** (from 14 fewer to 69 more)
**Intubation time**											
413 (5 RCTs)	serious	serious	not serious	not serious	none	⊕⊕◯◯ LOW	207	206	-	-	SMD **0.06 SD higher** (0.54 lower to 0.66 higher)
**Glottic view**											
739 (7 RCTs)	serious	serious	not serious	not serious	none	⊕⊕◯◯ LOW	325/369 (88.1%)	334/370 (90.3%)	**OR 0.91** (0.01 to 148.39)	881 per 1,000	**10 fewer per 1,000** (from 812 fewer to 118 more)

**Safety**											
689 (8 RCTs)	serious	not serious	not serious	serious	none	⨁⨁◯◯ LOW	277/344 (80.5%)	288/345 (83.5%)	**OR 1.13** (0.59 to 2.15)	805 per 1,000	**18 more per 1,000** (from 96 fewer to 94 more)
**GVL compared to MCL for Difficult airway**											
**Success**											
504 (7 RCTs)	serious	not serious	not serious	not serious	none	⨁⨁⨁◯ MODERATE	237/251 (94.4%)	240/253 (94.9%)	**OR 2.68** (1.23 to 5.81)	944 per 1,000	**34 more per 1,000** (from 10 more to 46 more)
**Intubation time**											
60 (1 RCT)	serious	not serious	not serious	not serious	none	⨁⨁⨁◯ MODERATE	30	30	-	-	SMD **0.6 SD lower** (1.12 lower to 0.08 lower)
**Glottic view**											
366 (4 RCTs)	serious	not serious	not serious	serious	none	⨁⨁◯◯ LOW	100/181 (55.2%)	171/185 (92.4%)	**OR 1.44** (0.00 to 1221.23)	552 per 1,000	**88 more per 1,000** (from -- to 447 more)
**Safety**	N/A										

CI: Confidence interval; **OR:** Odds ratio; **SMD:** Standardised mean difference; N/A: Not available
