# Supplementary material for: Comparisons of Videolaryngoscopes for Intubation Undergoing General Anesthesia: Systematic Review and Network Meta-Analysis of Randomized Controlled Trials
Source: J Pers Med. 2022 Feb 26;12(3):363. doi: 10.3390/jpm12030363 (PMC8954588; doi:10.3390/jpm12030363)
Supplement: Supplementary file 1 [file jpm-12-00363-s001.zip › Tables S2-S4.pdf]

**Table S2.** Analysis for factors attributing outcomes according to airway circumstances in included studies; All airway.

| No. | Author, year | Intubation time | Sedatives | Muscle relaxant | Intubator experience | ASA 1 | ASA 2 | ASA 3 | ASA 4 | ASA 5 |
|---|---|---|---|---|---|---|---|---|---|---|
| 1 | Ali 2012 | The time to secure the airway was defined as the time from introduction of the device between the incisors of the patient to connecting the endotracheal tube to the anaesthesia circuit. Correct placement of the tube was confirmed by the appearance of EtCO2 on the capnometer screen. | N/A | N/A | N/A | O | O | | | |
| 2 | Ali 2015 | Tracheal intubation duration was defined as the time between introduction of the device between the two incisors and appearance of the capnographic tracing | Propofol fentanyl | Succinylcholine | The anaesthetist in charge of the procedure was always the same and had training with both devices on 25 patients. | O | O | | | |
| 3 | Arici 2014 | The intubation time was defined as the time from the anesthesiologist taking the laryngoscope in his hand until the first upward deflection on the capnograph after the connection of the anesthetic ventilation system to the tracheal tube. | Thiopental sodium | Rocuronium | Anaesthesiologist | O | O | | | |
| 4 | Cavus 2011* | The time for endotracheal intubation was defined that the time from touching the tube to successful endotracheal placement. Correct tube position, and subsequently successful ventilation was assessed with capnography and bilateral chest auscultation. | Propofol Remifentanil | Rocuronium | One of three anaesthesiologists with at least eight years experience (after being trained on manikins with the CM) | O | O | O | | |
| 5 | Chalkeidis 2010* | The intubation procedure was started at this time and ended on the correct placement of the endotracheal tube. This was confirmed by a capnograph at the anesthesiologist's instruction. | Propofol Fentanyl | Cisatracurium | Four anesthesiologists were involved in this study. They were all consultants with similar experience and none of them had ever used the ATQ prior to this study. They each performed 15 intubations with the ATQ before data collection. | O | O | O | | |
| 6 | Kido 2015 | The time to DLT insertion was calculated from the time when the laryngoscope passed between the patient's lips until the confirmation | Propofol Remifentanil | Rocuronium | Anesthesia residents with 1-3 years of anesthesia training who had more than 50 experiences with DLT | O | O | O | | |

| No. | Author, year | Intubation time | Sedatives | Muscle relaxant | Intubator experience | ASA 1 | ASA 2 | ASA 3 | ASA 4 | ASA 5 |
|---|---|---|---|---|---|---|---|---|---|---|
| | | of end-tidal carbon dioxide on the capnograph. | | | intubation performed airway management. | | | | | |
| 7 | Kim 2013 | The duration of the first attempt and that of the successful attempt were recorded. Standard monitoring devices, including electrocardiography, noninvasive arterial pressure, pulse oximetry, and end-tidal concentrations of carbon dioxide. | Propofol Fentanyl | Rocuronium | Both anaesthetists experienced > 3 yr of clinical anaesthesia, and had performed >500 and at least 100 tracheal intubations with the MCL and the AWS in patients, respectively | O | O | | | |
| 8 | Lange 2009* | Insertion time measurement was performed by an independent observer and started after jaw opening when the optical laryngoscope passed the patient's lips and stopped when the tracheal tube passed the vocal cords. | Propofol Fentanyl | Succinylcholine | All tracheal intubations in the study were performed by three experienced (> 10 year) Board Certified anaesthesiologists who each had experience of at least 30 tracheal intubations with each device before starting the study. | O | O | O | | |
| 9 | Najafi 2014* | N/A | Thiopental | Atracurium | All patients were orally intubated after 3 minutes of induction by one anesthesiologist in both groups | O | O | | | |
| 10 | Ninan 2016* | The intubation time was defined as the interval from insertion of the laryngoscope blade into the mouth to inflation of the tracheal tube cuff. Following tracheal intubation, correct placement of tracheal tube was confirmed by auscultation and capnography. | Thiopentone | Vecuronium | The same anesthetist intubations in the study group. | O | O | | | |
| 11 | Nishikawa* 2009 | The duration of each attempt was recorded as the interval from the time the device was inserted into the oropharynx to the time when the device was removed from the oral cavity. Routine physiologic monitoring was commenced, and included electrocardiography, noninvasive arterial blood pressure, HR, SpO2, EtCO2 concentration, sevoflurane concentration, and body temperature. | Propofol Fentanyl | Vecuronium | All intubating procedures were performed by a single anesthesiologist who had 2 years experience with MCL and at least 50 times experience with the AWS. | O | O | | | |

| No. | Author, year | Intubation time | Sedatives | Muscle relaxant | Intubator experience | ASA 1 | ASA 2 | ASA 3 | ASA 4 | ASA 5 |
|---|---|---|---|---|---|---|---|---|---|---|
| 12 | Parasa 2016 | Intubation time was the time from introduction of the laryngoscope blade into the mouth to the visual appearance of EtCO2 trace the following intubation. | Midazolam, Fentanyl, Propofol | Vecuronium | All the intubations were performed by anesthesiology resident undergoing training, who have done a minimum of 50 intubations using MCL and 10 intubations using GVL. | O | O | | | |
| 13 | Sarkilar 2015* | Intubation time was defined that time when the laryngoscope passes through the patient's lips and when it is removed. | Sufentanil, Etomidate | Rocuronium | All intubations were performed by two anesthesiologists with experience in using VL. | N/A | N/A | N/A | N/A | N/A |
| 14 | Serocki 2013* | For each laryngoscopy, time was measured starting from touching the laryngoscope until achievement of best glottic view. Bilateral lung auscultation and capnography verified successful intubation and ventilation. | Propofol Remifentanil | Rocuronium | The investigation was carried out by three board certified anaesthesists familiar with all laryngoscopes (>50 intubations each). | O | O | O | | |
| 15 | Shah 2016 | The time taken for DLT insertion was calculated from the time the laryngoscope passed between the patient's lips until three complete cycles of end-tidal carbon dioxide were displayed on the capnography. | Propofol Fentanyl | Atracurium | All endotracheal intubations were performed by two experienced anaesthesiologists | O | O | | | |
| 16 | Sun 2005* | The time to intubation interval was measured from the time the instrument entered the patient's mouth until end-tidal carbon dioxide was detected. | Midazolam Fentanyl Propofol | Rocuronium Mivacurium | The intubations were performed by five different anaesthetists, all of whom were experienced in anaesthesia (>10 yr experience) and the use of the GVL (>20 intubations) prior to the study. | O | O | O | O | |
| 17 | Taylor 2013 | The time to intubation, defined as the time from the insertion of the laryngoscope into the oral cavity until its removal. Correct placement of the tracheal tube was confirmed by capnography and bilateral chest auscultation. | Propofol Fentanyl | Rocuronium | Each of the consultant anaesthetists involved in the study had previously practised with the MG using a manikin until subjectively comfortable with the device. | O | O | | | |
| 18 | Teoh 2009* | Time to successful tracheal intubation (defined as the interval from insertion of the laryngoscope 19blade into the mouth to the inflation of the endotracheal tube cuff) was recorded. | Propofol Fentanyl | Atracurium | All intubations were performed by experienced staff anaesthesiologists who had performed > 100 intubations with the GVL and > 30 intubations with AWS. | O | O | O | | |

| No. | Author, year | Intubation time | Sedatives | Muscle relaxant | Intubator experience | ASA 1 | ASA 2 | ASA 3 | ASA 4 | ASA 5 |
|---|---|---|---|---|---|---|---|---|---|---|
| 19 | Teoh 2010* | Time to successful tracheal intubation (defined as the interval from insertion of the laryngoscope blade into the mouth to inflation of the tracheal tube cuff) were recorded. | Propofol Fentanyl | Atracurium | All intubations were performed by experienced anaesthetists who had performed > 30 intubations with each of the devices being tested. | O | O | O | | |
| 20 | Wasem 2013* | The time taken to insert the tube began when the laryngoscope passed the patient's lips and ended when the tube passed the vocal cords, as announced verbally by the anaesthesiologist. | Propofol Fentanyl | Rocuronium | All tracheal intubations were performed by one of two permanent members of the thoracic anaesthesia team. Both operators had vast experience with the ATQ before conduction of the study. | O | O | O | | |
| 21 | Woo 2012* | The time taken for endotracheal intubation was recorded by an observer from the moment when the blade of the laryngoscope passed the incisor to the moment when it was outside the oral cavity after endotracheal intubation. | Propofol | Rocuronium | All endotracheal intubations were performed by a resident in the Department of Anesthesiology & Pain Medicine, with over 3 years of experience in endotracheal intubation using the MCL and with more than 50 procedures using the AWS. | | O | O | | |
| 22 | Yao 2015 | The time required for intubation with the double-lumen tube, measured by an independent observer as the time from when the laryngoscope blade was passed between the patient's lips until the first upstroke of the capnograph trace. | Sufentanil Propofol Remifentanil | Rocuronium | Laryngoscopy and intubation were performed by three senior anaesthetists with extensive experience of DLT placement using both MG and MCL. | O | O | O | | |
| 23 | Blajic 2019 | Time to tracheal tube placement was defined as the interval between insertion of the blade and detection of EtCO2 | Fentanyl Propofol | Suxamethonium Rocuronium | Laryngoscopy was attempted by one of the three attending anaesthesiologists, all of whom had performed >30 intubations with the respective devices. | | O | O | | |

Abbreviations: ASA, American Society of Anesthesiologists, N/A, not applicable; VL, videolaryngoscope; ETT, endotracheal tube; EtCO2, end tidal carbon dioxide; DLT, double lumen tube; MCL, Macintosh direct laryngoscope; ATQ, Airtraq video laryngoscope; AWS, Pentax Airwayscope; CM, C-MAC video laryngoscope; CMD, C-MAC D-Blade video laryngoscope; GVL, GlideScope; KV, King Vision video laryngoscope; MG, McGrath video laryngoscope.

*Excluded from meta-analysis of intubation time because the intubation success was not confirmed with capnography.

**Table S3.** Analysis for factors attributing outcomes according to airway circumstances in included studies; Normal airway.

| No. | Author, year | Intubation time | Sedatives | Muscle relaxant | Intubator experience | ASA 1 | ASA 2 | ASA 3 | ASA 4 | ASA 5 |
|---|---|---|---|---|---|---|---|---|---|---|
| 1 | Abdulmohsen 2016 | The time from holding of the investigated laryngoscope to the appearance of the first upward deflection on the capnography | Propofol Remifentanil | Rocuronium | Anesthesiologists who had limited experience in using the three VLs following receiving a simulation course contained education and training in using the studied VL | O | O | | | |
| 2 | Ahmed 2017 | The time taken from the insertion of the blade beyond the incisors until four square wave patterns of EtCO2 on the monitor. | Propofol | Vecuronium | The learning curve was achieved by the researcher with both the equipments by performing 20 intubations in mannequins followed by 10 intubations in patients before the commencement of study. | O | O | | | |
| 3 | Amini 2015 | The time from grasping the ETT until observing a square wave on the capnograph, was recorded | Propofol | Succinylcholine | A single experienced resident of anesthesiology. | O | O | | | |
| 4 | Aqil 2016 | The time of entry of the instrument into patient's oral cavity till detection of EtCO2 tracing on the monitor | Propofol fentanyl | Cisatracurium | Trainee residents having more than 1 year experience and who had successfully performed more than 50 tracheal intubations with each device. | O | O | | | |
| 5 | Aqil 2017 | N/A | Propofol fentanyl | Cisatracurium | Anesthetists | O | O | | | |
| 6 | Bakshi 2019 | The time from advancement of laryngoscope from dental arches to first deflection of capnograph. | Propofol fentanyl | Vecuronium | Eleven anaesthesiologists experienced with the use of MG | O | O | | | |
| 7 | Bhandari 2013 | The time elapsed from insertion of the blade between the dental arches until the ETT was placed through the vocal cords and confirmed by chest rise, auscultation, and square wave capnography | Propofol | Vecuronium | Anesthetists | O | O | | | |
| 8 | Bilehjani 2009* | The consumed time from opening of the mouth up to filling of the tube cuff. | Midazolam fentanyl | Cisatracurium | Anesthetists | O | O | O | | |
| 9 | Caparlar 2019 | The laryngoscope entering the mouth to removal with EtCO2 on the monitor. | - | - | Anesthetists | O | O | | | |
| 10 | ElTahan 2017 | N/A | Propofol fentanyl | Rocuronium | One of the two anesthetists (>4 years of experience) experienced in the use of each laryngoscope, | O | O | | | |

| No. | Author, year | Intubation time | Sedatives | Muscle relaxant | Intubator experience | ASA 1 | ASA 2 | ASA 3 | ASA 4 | ASA 5 |
|---|---|---|---|---|---|---|---|---|---|---|
| 11 | ElTahan 2018 | N/A | Propofol Remifentanil | Rocuronium | Anesthesia consultants, specialists, or trainees with at least 7 years, 3 years, or 6 months of clinical anesthesiology experience | | O | O | | |
| 12 | Hsu 2012 | The time when the laryngoscope passed between the patient's lips until three complete cycles of EtCO2 was displayed on the capnograph. | Thiamylal fentanyl | Rocuronium | Two experienced anaesthesiologists with experience of at least 300 tracheal intubations with each device. | O | O | | | |
| 13 | Jafra 2018 | From the insertion of device into the mouth to obtaining a square wave capnogram on monitor. | Propofol | Rocuronium | Laryngoscopist | O | O | | | |
| 14 | Jeon 2011 | The duration of one attempt was defined as the time elapsed between picking up the ETT and verification of tracheal intubation with visualization of three EtCO2 waveforms during mechanical ventilation | Propofol Remifentanil | Rocuronium | One of three anesthesiologists with extensive experience using the GVL and MG. | O | O | | | |
| 15 | Lee 2012 | N/A | Propofol fentanyl | Rocuronium | The staff members all received an introductory VL course in the hospital's airway skills lab and had used each of the VLs a minimum of 50 times prior to this study. | O | O | O | | |
| 16 | Lee 2013* | The time taken from when the tip of the blade passes the incisors until the time when the tip of the blade passes out of the incisors after inserting the ETT. | Midazolam, thiopental and fentanyl | Rocuronium | A single anesthesiologist who was an expert in both intubation procedures. | O | O | | | |
| 17 | Lee 2017 | The time from inserting the blade between the patient's incisors to the first appearance of EtCO2 on the capnograph | Propofol fentanyl | Rocuronium | Two anesthesiologists, both of whom had >10 years of clinical experience and had performed at least 20 successful intubations using both VL devices. | O | O | | | |
| 18 | Maharaj 2006* | The time taken from insertion of the blade between the teeth until the ETT was placed through the vocal cords, as confirmed visually by the anaesthetist. | Propofol fentanyl | atracurium | Four anaesthetists experienced in the use of both laryngoscopes. | O | O | O | | |

| No. | Author, year | Intubation time | Sedatives | Muscle relaxant | Intubator experience | ASA 1 | ASA 2 | ASA 3 | ASA 4 | ASA 5 |
|---|---|---|---|---|---|---|---|---|---|---|
| 19 | Maruyama 2008* | The time when the MCL or the AWS passed the central incisors to the time when the tip of the ETT passed through the glottis. | Propofol or ketamine and fentanyl | Vecuronium | Anesthetists | O | O | | | |
| 20 | Maruyama 2011* | The time elapsed from insertion of the laryngoscope until removal from the oral cavity. | Propofol fentanyl | Rocuronium | Five anesthesiologists having combined experience of more than 400 intubations using the MCL and more than 40 intubations with the AWS/ | O | O | | | |
| 21 | Mendonca 2018 | N/A | Fentanyl or remifentanil and propofol | Atracurium or rocuronium | Three investigators, experienced in the use of both VLs (more than 50 intubations with each device) | O | O | O | | |
| 22 | Pazur 2016 | The time the laryngoscope was taken into the hand until the EtCO2 curve was displayed on the monitor. | Sufentanyl, propofol 2 | Rocuronium | Anesthetists | O | O | | | |
| 23 | Raza 2017* | The time from insertion of the blade between the teeth until the ETT was placed through the vocal cords, as evidenced by visual confirmation by the anesthetist. | Propofol | Vecuronium | The anomalies of learning curve for the use of the equipment were decreased using each equipment for tracheal intubation 20 times in manikin on separate occasions followed by ten tracheal intubations in patients in the operation theater before starting the study. | O | O | | | |
| 24 | Russell 2013* | The time from endobronchial intubation until fibreoptic bronchoscopic confirmation of adequate placement. | Anaesthesia was induced using medications and dosages at the anaesthetist's discretion. | Anaesthesia was induced using medications and dosages at the anaesthetist's discretion. | All anaesthetists who took part in this study were specialists or fellows who regularly perform thoracic anaesthesia and regularly use the GVL for tracheal intubation. | N/A | | | | |
| 25 | Sargin 2016 | N/A | Propofol fentanyl | Rocuronium | One anesthesiologist who had used MG and MCL more than 50 times clinically. | O | O | | | |
| 26 | Siddiqui 2009* | The time from insertion of the intubating device into the oral cavity to inflation of the endotracheal tube cuff | Midazolam Fentanyl Propofol | Rocuronium | Anaesthesiologist, who had performed more than 50 intubations with MCL and GVL and is well experienced in all two techniques of tracheal intubations | O | O | | | |
| 27 | Tempe 2016 | The time interval between oral | Midazolam | Pancuronium | Senior experienced anesthetists | O | O | O | O | |

| No. | Author, year | Intubation time | Sedatives | Muscle relaxant | Intubator experience | ASA 1 | ASA 2 | ASA 3 | ASA 4 | ASA 5 |
|---|---|---|---|---|---|---|---|---|---|---|
| | | placement of the ETT to the attainment of tracing of 3 EtCO2 waveforms after intubation and initiation of mechanical ventilation. The duration of laryngoscopy and intubation was defined as the sum of all intubation attempts. | Fentanyl Thiopentone | | who had experience of at least 50 intubations using VL in mannikin and at least 20 in patients. | | | | | |
| 28 | Turkstra 2016 | The time from when the facemask was removed from the patient's face to when EtCO2 of at least 20 mm Hg was measured on the end-tidal gas monitor | N/A | Rocuronium. | Staff anesthesiologists and experienced anesthesiology residents (postgraduate year 3 or above) who have performed at least 3 intubations. | O | O | O | O | |
| 29 | Wan 2016 | The intubation time, measured as the time from the insertion of the VL until the completion of intubation using capnography by an independent observer. | Propofol | Rocuronium | Experienced anesthesiologists have ever performed DLT intubation in >200 cases with MCL, and in 30 cases with either MG or ATQ. | O | O | O | | |
| 30 | Yi 2015 | The intubation time was defined as the time period between the laryngoscopes passed the patient's lips and the completion of the tube advancement into the trachea. ETT placement was confirmed by capnography. | Propofol Fentanyl | Rocuronium | Senior anesthesiologist with experience in more than 30 DLT intubation cases with the ATQ and the GVL. | O | O | | | |
| 31 | Varsha 2019 | The time from insertion of insertion of laryngoscope into mouth to obtaining three capnographs waveforms after intubation | Midazolam Fentanyl Sevoflurane | Vecuronium | Anesthesiologists who had more than 7-year experience with MCL and had done 20 intubations with ATQ. | | | O | | |
| 32 | Tosh 2018 | N/A | Propofol Isoflurane | Vecuronium | Anesthetists | O | O | | | |
| 33 | Reena 2019 | The time interval between passing the scope's blade through interdental line to confirmation of correct ETT placement by capnography. | Fentanyl Propofol | Vecuronium | Anesthesiologist with at least 5 years of experience and 50 intubations using KV | O | O | | | |

Abbreviations: ASA, American Society of Anesthesiologists; N/A, not applicable; VL, videolaryngoscope; ETT, endotracheal tube; EtCO2, end tidal carbon dioxide; DLT, double lumen tube; MCL, Macintosh direct laryngoscope; ATQ, Airtraq video laryngoscope; AWS, Pentax Airwayscope; CM, C-MAC video laryngoscope; CMD, C-MAC D-Blade video laryngoscope; GVL, GlideScope; KV, King Vision video laryngoscope; MG, McGrath video laryngoscope.

*Excluded from meta-analysis of intubation time because the intubation success was not confirmed with capnography.

**Table S4.** Analysis for factors attributing outcomes according to airway circumstances in included studies; Difficult airway.

| No. | Author, year | Intubation time | sedatives | Muscle relaxant | Intubator experience | ASA 1 | ASA 2 | ASA 3 | ASA 4 | ASA 5 |
|---|---|---|---|---|---|---|---|---|---|---|
| 1 | Abdallah 2011 | The time from start of the first attempt of the insertion of the laryngoscope until a capnogram signal was obtained. | N/A | N/A | Attending anesthesiologists whom had previously used the AWS 5 to 10 times before the study began. |  | O | O | O |  |
| 2 | Akbar 2015 | The time when either of the blades was inserted beyond the lip until first appearance of the capnograph waveform | Propofol Fentanyl Sevoflurane | Rocuronium | Anesthetic trainee whose previous experience includes >30 intubations with the CM and more than 5 years frequent use of the MCL. | O | O |  |  |  |
| 3 | Akbas 2019 | The time from when the anesthesiologist picked up the VL to when the anesthesiologist successfully placed the ETT through the vocal cords, which was assessed by the detection of meaningful EtCO2 levels using capnography. | Propofol Fentanyl | Rocuronium | Anesthesiologists whom had >7 years of clinical experience and had performed at least 100 successful intubations using both VLs. |  |  | O |  |  |
| 4 | Ali 2017 | The time from insertion of the laryngoscope to confirmation of intubation by capnography | Propofol | Succinylcholine | Experienced anaesthesiologist whose previous experience included more than 20 intubations with each laryngoscope. | O | O |  |  |  |
| 5 | Ander 2017 | The time from the moment the anesthetist took the laryngoscope handle until EtCO2 was registered on the ventilator monitor | Propofol Remifentanil | Rocuronium | Anesthetists experienced with both devices used in the study (>50 intubations with each device). | O | O | O |  |  |
| 6 | Aoi 2010 | The time when the airway device was handed to the anesthesiologist to the time when the presence of EtCO2 was confirmed in the exhaled breath on the vital sign monitor. | Fentanyl Propofol Sevoflurane | Vecuronium | Anesthesiologist experienced in the use of both devices | O | O |  |  |  |
| 7 | Belze 2017 | The time from when the VL entered between the patient's lips until confirmation of successful tracheal intubation by the three capnograms | Sufentanil Propofol | Rocuronium | Anaesthesiologists who had inserted at least 10 DLTs with each device on a manikin devoted to normal intubation training | O | O | O | O |  |
| 8 | Bruck 2015* | The time between the | Propofol or | Rocuronium or | Senior attending | O | O | O |  |  |

| No. | Author, year | Intubation time | sedatives | Muscle relaxant | Intubator experience | ASA 1 | ASA 2 | ASA 3 | ASA 4 | ASA 5 |
|---|---|---|---|---|---|---|---|---|---|---|
| | | laryngoscope's passing the patients' lips to the ETT's passing the vocal cords, as announced verbally by the anaesthetist. | Etomidate and Fentanyl | Cisatracurium | anaesthesiologists working in the neurosurgery anaesthesia section | | | | | |
| 9 | Chandrashekaraiah 2017 | N/A | Fentanyl Propofol Sevoflurane. | Atracurium | Experienced anaesthetist | O | O | | | |
| 10 | Dhonneur 2009 | The time elapsing from oral insertion of the airway to the first optimal ventilation cycle as confirmed by EtCO2 monitoring after ETT cuff inflation | Sufentanil Propofol | Succinylcholine | Skilled senior anesthesiologists. | O | O | O | | |
| 11 | Enomoto 2008 | For the MCL, time was taken from a tracheal tube passing the gap between the upper and the lower incisors to confirmation of EtCO2 waveforms after tracheal intubation. For the AWS, time was taken from touching the ETT (attached to the scope) to confirmation of EtCO2 waveforms after tracheal intubation. | Propofol Sevoflurane | Vecuronium | Anaesthetist | O | O | | | |
| 12 | Foulds 2016 | This time, a ETT was passed into the glottis, and the time taken for the intubation (from the passage of the tip of the laryngoscope past the patient's teeth to the appearance of EtCO2 on the capnograph trace) | Intravenous access was obtained, and anaesthesia was induced (and maintained) according to the investigator's preference. | Intravenous access was obtained, and anaesthesia was induced (and maintained) according to the investigator's preference. | Anaesthetist | O | O | O | | |
| 13 | Gupta 2013 | The time taken from insertion of the laryngoscope blade between the teeth until the ETT is placed through the vocal cords, as evidenced by visual confirmation. If the passage of ETT was not directly visualized, the intubation attempt was considered complete after ETT was connected to the anesthetic circuit and a capnography trace was obtained. | Propofol Fentanyl | Rocuronium | Anesthesiologists experienced in the use of both laryngoscopes in patients requiring manual in-line stabilization, having done more than 50 such intubations with each device before this study. | O | O | O | | |
| 14 | Hosalli 2017 | N/A | Propofol | Vecuronium | Anaesthesiologist who was experienced in using all three laryngoscopes. | O | O | | | |

| No. | Author, year | Intubation time | sedatives | Muscle relaxant | Intubator experience | ASA 1 | ASA 2 | ASA 3 | ASA 4 | ASA 5 |
|---|---|---|---|---|---|---|---|---|---|---|
| 15 | Hu 2017* | The intubation time was measured from the laryngoscope passing the patient's lips until the ETT was deemed to be correctly positioned by each participant. | Propofol Sufentanil Remifentanil | Cisatracurium | Senior anesthesiologist who had experience with using GVL and MCL in more than 100 intubation cases. | O | O | | | |
| 16 | Ilyas 2014 | The time from when the allocated laryngoscope entered the mouth to when the first capnographic square wave was completed. | Propofol | Rocuronium | The anaesthetists performing intubation had a minimum of 10 years' experience and were employed in major tertiary trauma centres. | O | O | O | | |
| 17 | KleineBrueggeney 2016 | Success of the first intubation attempt was the primary outcome parameter. Success was defined as placement of the ETT in the trachea with-in 180 s, confirmed by EtCO2. | Propofol Fentanyl | Rocuronium | All participating consultant anaesthetists were airway management experts and trained with all VLs on both manikins and patients until they, as airway specialists, felt competent with each device | O | O | O | | |
| 18 | KleineBrueggeney 2017 | Time was measured from taking the face-mask away from the face until appearance of EtCO2. | Propofol Fentanyl | Rocuronium | All participating consultant anaesthetists were airway management experts and trained with all VLs on manikins and patients until they felt competent with each device. The level of experience was the same with all VLs and none of the devices had been a standard intubation tool before the study start except for the standard MCL. | O | O | O | | |
| 19 | Lim 2005 | The anaesthetist then introduced the ETT with stylet and correct placement was confirmed with capnography. Time taken for successful tracheal intubation: measured from the time the anaesthetist picked | Propofol Fentanyl | Atracurium | Twenty anaesthetists in our department with more than two years of anaesthetic training participated in this trial. The anaesthetists' experience with the GVL ranged from being a novice user | O | O | | | |

| No. | Author, year | Intubation time | sedatives | Muscle relaxant | Intubator experience | ASA 1 | ASA 2 | ASA 3 | ASA 4 | ASA 5 |
|---|---|---|---|---|---|---|---|---|---|---|
| | | up the assigned intubating device to the time capnography confirmed the correct placement of the ETT. | | | to having had more than 10 successful tracheal intubations using the GVL. The anaesthetist's level of experience with the GVL (Level 0: no previous experience with the GVL, Level 1: 1-5 previous tracheal intubations with the GVL, Level 2: 6-10 previous tracheal intubations with the GVL and Level 3: >10 previous tracheal intubations with the GVL). | | | | | |
| 20 | Liu 2009 | The time for successful tracheal intubation, measured from the time the face mask was lifted off the face to the time of the first breath after tracheal intubation, with correct intubation and ventilation confirmed by EtCO2 capnography. This timing did not include the time taken for direct laryngoscopy with the MCL. | Propofol Fentanyl | Atracurium | The investigators had moderate experience with both VLs. It was not possible to completely standardize their experience with both devices, and the GVL had been in use for a longer time in our institution. | O | O | O | | |
| 21 | Maharaj 2007* | The duration of the intubation attempt was defined as the time taken from insertion of the blade between the teeth until the ETT was placed through the vocal cords, as evidenced by visual confirmation by the anesthesiolo-gist. | Fentanyl Propofol | Atracurium | One of four anesthesiologists experienced in the use of both laryngoscopes. | O | O | O | | |
| 22 | Maharaj 2008* | The duration of the intubation attempt was defined as the time taken from insertion of the blade between the teeth until the tracheal tube was placed through the vocal cords, as | Fentanyl propofol | Atracurium | Laryngoscopy was performed by one of three anaesthetists experienced inthe use of both laryngoscopes. Each | O | O | O | | |

| No. | Author, year | Intubation time | sedatives | Muscle relaxant | Intubator experience | ASA 1 | ASA 2 | ASA 3 | ASA 4 | ASA 5 |
|---|---|---|---|---|---|---|---|---|---|---|
| | | evidenced by visual confirmation by the anaesthetist. | | | anaesthetist had performed >500 intubations using the MCL, and at least 50 intubations with the ATQ in manikins, and 50 intubations with the ATQ in patients, prior to this study. | | | | | |
| 23 | Malik 2008* | The time taken from insertion of the blade between the teeth until the tracheal tube was placed through the vocal cords, as evidenced by visual confirmation by the anaesthetist. | Fentanyl Propofol | Atracurium | Each investigator had performed at least 50 intubations with each device in mani- kins, and at least 20 intubations in the clinical setting with each device | O | O | O | | |
| 24 | Malik1 2009* | The duration of the intubation attempt was defined as the time taken from insertion of the blade between the teeth until the ETT was placed through the vocal cords, as evidenced by visual confirmation by the anaesthetist. | Fentanyl Propofol | Atracurium | Each investigator had performed, with each device, at least 50 intuba- tions in manikins and at least 20 intubations in the clinical setting. | O | O | O | | |
| 25 | Malik2 2009* | The duration of the intubation attempt was defined as the time taken from insertion of the blade between the teeth until the ETT was placed through the vocal cords, as evi- denced by visual confirmation by the anaesthetist perform- ing laryngoscopy. | Fentanyl Propofol | Atracurium | Each anaesthetist had performed more than 500 intubations with the MCL and at least 100 intubations with the AWS and GVL in manikins, and 50 intubations with the AWS and GVL in patients, before this study. | O | O | O | | |
| 26 | Mathew 2018 | The time taken from the tube insertion to the properly placement with showing capnogram traces | Fentanyl Propofol | Vecuronium | All intubations were performed by anaesthesiologists with more than 2-years of experience with the MCL and ATQ | O | O | | | |
| 27 | McElwain 2011* | The time taken from when the anaesthetist | Fentanyl Propofol | Unknown NMB | The trachea was then intubated by one | O | O | O | | |

| No. | Author, year | Intubation time | sedatives | Muscle relaxant | Intubator experience | ASA 1 | ASA 2 | ASA 3 | ASA 4 | ASA 5 |
|---|---|---|---|---|---|---|---|---|---|---|
| | | indicated the best view at laryngoscopy until the ETT was placed through the vocal cords, as evidenced by visual confirmation by the anaesthetist. | | | anaesthetist experienced in the use of all three laryngoscopes. | | | | | |
| 28 | Nandakumar 2018 | the time taken from the time when the blade of the laryngoscope crosses the incisors to the first upstroke of the capnograph | Fentanyl Propofol | Succinylcholine | Senior anesthesiologist. | O | O | O | | |
| 29 | Ndoko 2008* | Tracheal intubation duration was defined as the time elapsing between the insertion of the laryngoscope into the oral cavity and the visualization (or the sensation in the case of blind tracheal intubation) of the ETT crossing the glottis. | Sufentanil propofol | Succinylcholine Atracurium | All anaesthesiologists performing tracheal intubations were skilled in the use of the ATQ and MCL and frequently anaesthetize for obesity surgery. | O | O | O | | |
| 30 | Ng 2012 | The time taken for successful intubation, defined as time from when the allocated VL was inserted into the participant's mouth until EtCO2 was detected. | Anaesthesia was induced using the drugs of the treating anaesthetist's choice. | Anaesthesia was induced using the drugs of the treating anaesthetist's choice. | Laryngoscopy was performed with either the MG or the CM (blade size 3 or 4) by an anaesthetist with a minimum of 10-year experience and who had used both devices at least 10 times clinically. Both the MG and the CM had been available for over a year before the commencement of the study. | O | O | O | | |
| 31 | Ranieri 2012 | Time in seconds from the moment the anaesthetist picked up the device until cuff inflation using capnography | Fentanyl Propofol | Suxamethonium Vecuronium | The four participating anaesthetists had more than 5-years of clinical experience with MCL and had used the ATQ on manikins and for a minimum of 50 patients. | O | O | O | | |
| 32 | Sahajanandan 2019 | "Time for intubation" is time taken from insertion of blade into the patients' mouth till three $ETCO_2$ tracings were seen on the monitor. | Fentanyl Propofol | Rocuronium | All anesthetists who intubated, performed 20 intubations with both VLs on manikin before | O | O | | | |

| No. | Author, year | Intubation time | sedatives | Muscle relaxant | Intubator experience | ASA 1 | ASA 2 | ASA 3 | ASA 4 | ASA 5 |
|---|---|---|---|---|---|---|---|---|---|---|
| | | | | | performing the study case. | | | | | |
| 33 | Shravanalakshmi 2017* | Time from passing of the blade through teeth to passing of the ET tube beyond glottis | Fentanyl Propofol | Vecuronium | All the intubations were performed (as per the manufacturer recommendations) by an experienced anesthesiologist, who had done at least 30 intubations, with each device. | O | O | | | |
| 34 | Toker 2019 | The time measured from the point at which the laryngoscope blade was inserted into the mouth, and timing stopped upon the detection of an EtCO2 trace. | Propofol | Rocuronium | Experienced anesthesiologist in airway management and using VLs | | O | | | |
| 35 | Tolon 2012 | The time taken from insertion of the blade of the laryngoscope between the teeth until the endo-tracheal tube (ETT) is passed through the vocal cords and confirmed by capnography | Fentanyl Propofol | Atracurium | Experienced anesthetist | O | O | | | |
| 36 | Vijayakumar 2016 | Intubation time was defined as time from introduction of the laryngoscope into the oral cavity till confirmation of intubation by capnogram. | Fentanyl Propofol | Vecuronium | One of the two experienced anesthesiologists performed intubations. These anesthesiologists were initially trained in manikins and have been routinely using ATQ clinically. They have intubated >100 patients with normal airway and >20 anticipated difficult airways using ATQ before the study. | O | O | | | |
| 37 | Wasinwong 2017 | Intubation time was defined as time from introduction of the laryngoscope into the oral cavity till confirmation | Propofol | Succinylcholine | Second year anesthesia residents who had experience in the use of GVL at least 10 times | O | O | O | | |

| No. | Author, year | Intubation time | sedatives | Muscle relaxant | Intubator experience | ASA 1 | ASA 2 | ASA 3 | ASA 4 | ASA 5 |
|---|---|---|---|---|---|---|---|---|---|---|
| | | of intubation by capnogram | | | | | | | | |
| 38 | Xue 2007* | The period from termination of manual ventilation using a facemask to restarting of ventilation through a ETT | Fentanyl Propofol | Vecuronium | a single anesthesiologist experienced in using a MCL and a GVL. | O | | | | |
| 39 | Yoo 2018 | The time from when the laryngoscope passed between the patient's lips to the confirmation of EtCO2 on the capnograph | Remifentanyl Propofol | Rocuronium | An experienced anesthetist for MG and MCL | O | O | | | |
| 40 | Yumul 2016 | Times from the passage of the blade between the teeth to obtain glottic view, to placement of the tracheal tube and the appearance of an EtCO2 waveform | Lidocaine Propofol | Succinylcholine | All anesthesiologists performing the tracheal intubations had been previously trained using all VLs with a minimum of 20 intubations with each device | O | O | O | | |

Abbreviations: ASA, American Society of Anesthesiologists; N/A, not applicable; VL, videolaryngoscope; ETT, endotracheal tube; EtCO2, end tidal carbon dioxide; DLT, double lumen tube; MCL, Macintosh direct laryngoscope; ATQ, Airtraq video laryngoscope; AWS, Pentax Airwayscope; CM, C-MAC video laryngoscope; GVL, GlideScope; KV, King Vision video laryngoscope; MG, McGrath video laryngoscope; NMB, neuromuscular blocking agent

*Excluded from meta-analysis of intubation time because the intubation success was not confirmed with capnography.
